# Cancer Risk and Estimated Lithium Exposure in Drinking Groundwater in the US

**DOI:** 10.1001/jamanetworkopen.2024.60854

**Published:** 2025-02-20

**Authors:** Jiajun Luo, Liang Zheng, Zhihao Jin, Yuqing Yang, William Isaac Krakowka, Eric Hong, Melissa Lombard, Joseph Ayotte, Habibul Ahsan, Jayant M. Pinto, Briseis Aschebrook-Kilfoy

**Affiliations:** 1 Department of Public Health Sciences, The University of Chicago Biological Science Division, Chicago, Illinois; 2 Institute for Population and Precision Health, The University of Chicago Biological Science Division, Chicago, Illinois; 3 Department of Thyroid Surgery, The First Hospital Affiliated With Sun Yat-Sen University, Guangzhou, China; 4 Gangarosa Department of Environmental Health, Rollins School of Public Health, Emory University, Atlanta, Georgia; 5 New England Water Science Center, US Geological Survey, Pembroke, New Hampshire; 6 Department of Family Medicine, The University of Chicago Biological Science Division, Chicago, Illinois; 7 Department of Surgery, The University of Chicago Biological Science Division, Chicago, Illinois

## Abstract

**Importance:**

Lithium is a naturally occurring element in drinking water and is commonly used as a mood-stabilizing medication. Although clinical studies have reported associations between receiving lithium treatment and reduced cancer risk among patients with bipolar disorder, to our knowledge, the association between environmental lithium exposure and cancer risk has never been studied in the general population.

**Objectives:**

To evaluate the association between exposure to lithium in drinking groundwater and cancer risk in the general population.

**Design, Setting, and Participants:**

This cohort study included participants with electronic health record and residential address information but without cancer history at baseline from the All of Us Research Program between May 31, 2017, and June 30, 2022. Participants were followed up until February 15, 2023. Statistical analysis was performed from September 2023 through October 2024.

**Exposure:**

Lithium concentration in groundwater, based on kriging interpolation of publicly available US Geological Survey data on lithium concentration for 4700 wells across the contiguous US between May 12, 1999, and November 6, 2018.

**Main Outcome and Measures:**

The main outcome was cancer diagnosis or condition, obtained from electronic health records. Stratified Cox proportional hazards regression models were used to estimate the hazard ratios (HRs) and 95% CIs for risk of cancer overall and individual cancer types for increasing quintiles of the estimated lithium exposure in drinking groundwater, adjusting for socioeconomic, behavioral, and neighborhood-level variables. The analysis was further conducted in the western and eastern halves of the US and restricted to long-term residents living at their current address for at least 3 years.

**Results:**

A total of 252 178 participants were included (median age, 52 years [IQR, 36-64 years]; 60.1% female). The median follow-up time was 3.6 years (IQR, 3.0-4.3 years), and 7573 incident cancer cases were identified. Higher estimated lithium exposure was consistently associated with reduced cancer risk. Compared with the first (lowest) quintile of lithium exposure, the HR for all cancers was 0.49 (95% CI, 0.31-0.78) for the fourth quintile and 0.29 (95% CI, 0.15-0.55) for the fifth quintile. These associations were found for all cancer types investigated in both females and males, among long-term residents, and in both western and eastern states. For example, for the fifth vs first quintile of lithium exposure for all cancers, the HR was 0.17 (95% CI, 0.07-0.42) in females and 0.13 (95% CI, 0.04-0.38) in males; for long-term residents, the HR was 0.32 (95% CI, 0.15-0.66) in females and 0.24 (95% CI, 0.11-0.52) in males; and the HR was 0.01 (95% CI, 0.00-0.09) in western states and 0.34 (95% CI, 0.21-0.57) in eastern states.

**Conclusions and Relevance:**

In this cohort study of 252 178 participants, estimated lithium exposure in drinking groundwater was associated with reduced cancer risk. Given the sparse evidence and unknown mechanisms of this association, follow-up investigation is warranted.

## Key Points

**Question:**

Is lithium exposure in drinking water associated with cancer risk in the general US population?

**Findings:**

In this cohort study of 252 178 individuals from the All of Us Research Program, higher lithium exposure in drinking groundwater was associated with reduced risk of all cancer types investigated overall and stratified by females and males.

**Meaning:**

Exposure to a higher estimated lithium level in drinking water may be associated with a lower incidence of cancers.

## Teaser

This cohort study uses data from the All of Us Research Program to examine associations between lithium levels in drinking groundwater across the US and risk of overall and major types of cancer in the general population.

## Introduction

Lithium is a naturally occurring element and can be commonly found in groundwater, particularly in arid areas in the western US, leading to widespread exposure through drinking water among the general population.^1^ As lithium demonstrates mood-stabilizing effects,^2^ it has been extensively used as a psychiatric medication for bipolar disorder, schizophrenia, and depression.^3,4,5,6^ The toxic serum level for lithium is close to its therapeutic level.^7^ Researchers have documented long-term adverse effects of lithium treatment on kidney and thyroid functions.^8,9^ However, several observational studies among patients with bipolar disorder have reported an association between receiving lithium treatment and reduced cancer risk.^10,11,12^ At the biological level, lithium has also been found to affect several enzymes that are implicated in cancer progression,^13^ such as glycogen synthase kinase-3^14^ and inositol monophosphate,^15^ providing a plausible biological basis for potential anticancer effects.

Despite these limited insights into the health impact of lithium, the broader health implications of environmental lithium exposure, with a demonstrated level substantially lower than typical medication doses, remain inadequately understood among the general population. Prior investigation of environmental lithium exposure, which was generally limited to psychotic and mental health outcomes, such as dementia,^16,17^ suicide,^18,19^ psychotic experiences,^20^ and crime rate^21^ or homicide incidence,^22^ generally indicated an association of lithium exposure with lower risk of these outcomes. However, a recent study found that higher lithium levels in drinking water among pregnant women were associated with an increased risk of autism among offspring,^23^ and a large-scale Swedish study concluded that lithium in drinking water was associated with increased risk of schizophrenia spectrum disorder.^24^ Research on other health outcomes is scarce, with 1 study concluding that environmental lithium exposure during pregnancy may lead to reduced fetal size.^25^ Other health outcomes, particularly chronic diseases such as cancer, are underinvestigated in relation to environmental lithium exposure. Currently, lithium is on the US Environmental Protection Agency (EPA) contaminant candidate list^26^ but is not subject to any proposed or promulgated regulations. Exposure to increasing environmental lithium contamination through the use and improper disposal of lithium-containing products (eg, medication, rechargeable batteries, and e-waste) has raised concerns. In light of growing concerns and preliminary findings, the EPA announced initiatives in October 2023 to gather more comprehensive data on the health outcomes associated with environmental lithium exposure in drinking water.^27^ Given the potential role of lithium in carcinogenesis, we conducted this nationwide epidemiologic study to investigate the association between lithium exposure in drinking groundwater across the contiguous US and cancer risk in the general population based on electronic health record (EHR) data of All of Us Research Program participants.^28^

## Methods

### Study Population and Outcome Assessment

This cohort study adhered to the Strengthening the Reporting of Observational Studies in Epidemiology (STROBE) reporting guideline. The study population included All of Us participants with valid EHR and residential address information between May 31, 2017, and June 30, 2022, and released as of February 15, 2023. The All of Us Research Program, initiated in 2017, is a prospective cohort that currently includes more than 544 000 adults living in the US. The goals, recruitment methods and sites, and scientific rationale for All of Us are described elsewhere.^28^ All of Us data include participants’ responses to a series of questionnaires, physical measurements collected by study staff at enrollment, and information from participants’ EHRs. The data are made available to researchers via the All of Us Researcher Workbench. This study was overseen and approved by the All of Us institutional review board. Informed consent was waived because only deidentified archival data were used.

We excluded individuals who had any cancer history prior to enrollment in the EHR or self-reported surveys. Cancer incidence was identified using the primary diagnosis or condition in the EHR. We excluded individuals who only had records of secondary cancers or metastatic cancers but no information on the primary cancer in the EHR. The observation period was defined as the period between enrollment and the date of the initial cancer diagnosis or condition after enrollment, death, or February 15, 2023, whichever occurred first. Covariates retrieved and adjusted in this study can be found in eMethods 1 in Supplement 1.

### Lithium Exposure Assessment

This study used data on groundwater used for drinking water supply from nation-scale studies implemented by the US Geological Survey (USGS).^1^ The data are from measured lithium concentrations in untreated groundwater from 1464 public-supply wells, 1676 domestic-supply wells, and 1560 shallow monitoring wells (4700 total) across the contiguous US that were sampled between May 12, 1999, and November 6, 2018 (eFigure 1 in Supplement 1). The median lithium concentration of these wells was 5.4 μg/L, with a range from less than 1 to 1700 μg/L. The lithium exposure data are available in the USGS data release.^29^

We used kriging to estimate the lithium concentration at the 1 km × 1 km grid level based on weighted averages of surrounding measurements, as suggested by previous studies.^16,23^ This method assumed that lithium concentrations are spatially autocorrelated (ie, lithium concentration at a certain location is closer to that at a nearby location than at distant ones). Details can be found in eMethods 2 in Supplement 1. The final kriging map of lithium concentrations can be found in eFigure 1 in Supplement 2.

**Figure 1.**
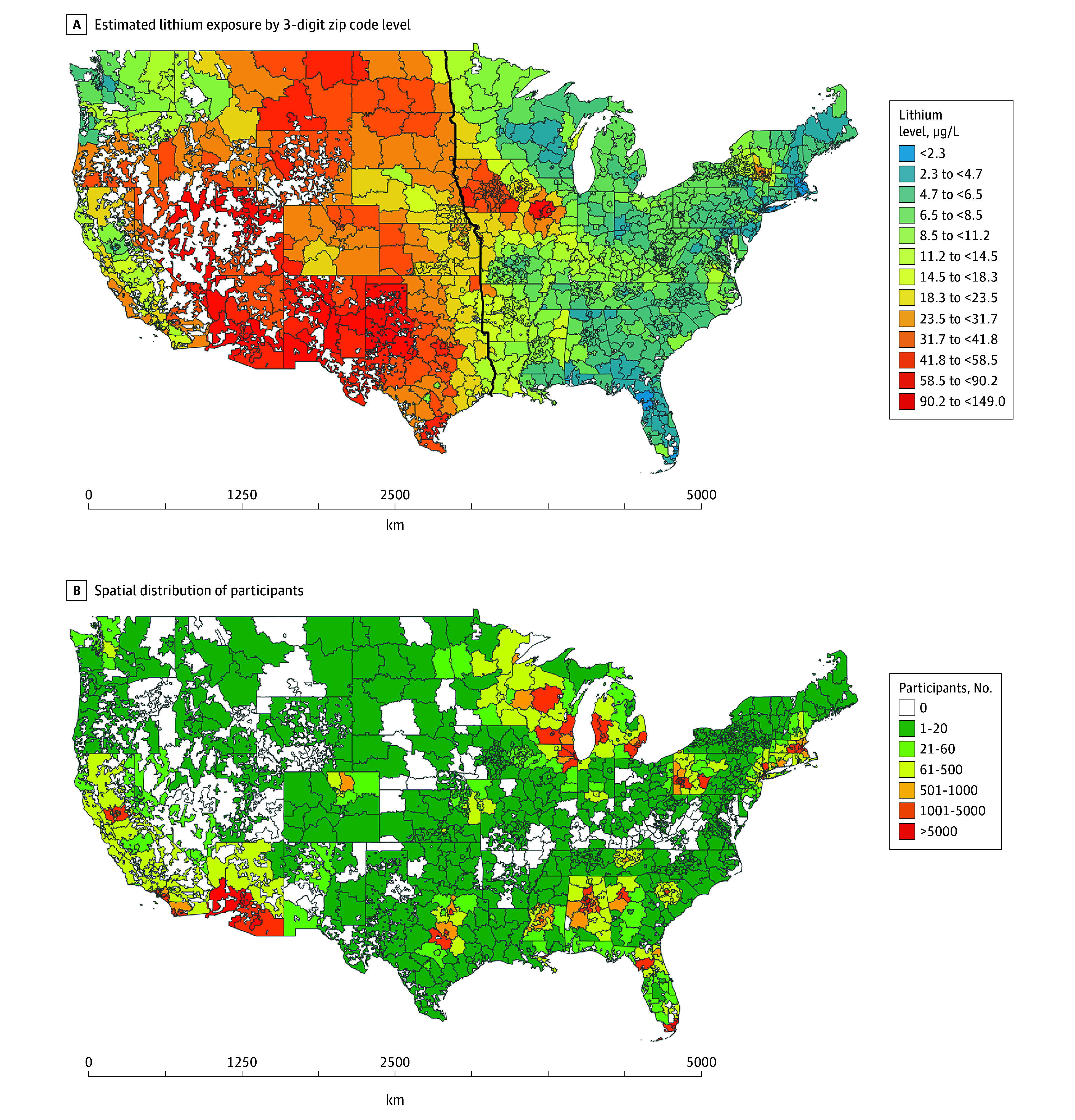
Estimated Lithium Exposure From Drinking Groundwater and Distribution of All of US Participants in the Contiguous US A, Black line indicates the boundary between western and eastern US regions.

Lithium exposure for each participant was assigned based on the 3-digit zip code of the residential address collected at enrollment, the only address information as of March 2024. We averaged the lithium concentration across all 1 km × 1 km grids within the 3-digit zip code and used the mean level as the exposure level. The spatial distributions of lithium exposure and the study population can be found in Figure 1.

### Statistical Analysis

We used stratified Cox proportional hazards regression models to estimate the hazard ratio (HR) and 95% CI for cancer in association with estimated lithium exposure. The estimated lithium exposure was categorized into 5 groups based on quintiles (quintile 1, 1.3-3.6 μg/L; quintile 2, 3.7-6.1 μg/L; quintile 3, 6.2-7.2 μg/L; quintile 4, 7.3-25.5 μg/L; and quintile 5, 25.6-149.9 μg/L), with the first quintile as the reference. The stratified terms included sex assigned at birth, race and ethnicity, and age at enrollment (10-year intervals). Sex was ascertained from the EHR and included female, male, and other (other was reported in the database but not otherwise specified). Race and ethnicity were ascertained by self-report and included in the analysis to assess racial disparities in associations between environmental exposures and health outcomes. Categories were Hispanic or Latino, non-Hispanic Black (hereafter, *Black*), non-Hispanic White (hereafter, *White*), and other (included American Indian, Asian, multiracial, or other than those listed). The models were additionally adjusted for educational level, household income, smoking status, alcohol drinking status, and the Deprivation Index at the residential address. To adjust for residual autocorrelation within the geographic units (ie, 3-digit zip code), we used the generalized estimating equation to calculate a statistically robust 95% CI. To estimate the nonlinear exposure-response curve, we fitted stratified Cox proportional hazards regression models with penalized splines for lithium exposures without specifying preset parameters for the spline. Missing values were addressed using multiple imputation based on the random forest imputation algorithm.^30^ We imputed 5 complete datasets and pooled estimates from these datasets according to the Rubin rule. All models followed these criteria.

We ran the regression model for overall cancer risk in the entire population and in sex-stratified populations. Moreover, we ran individual regression models for major cancer types, including female breast cancer, male prostate cancer, bladder and urinary cancer, central nervous system (CNS) cancer, colorectal cancer, kidney cancer, leukemia, non-Hodgkin lymphoma (NHL), and thyroid cancer.

Eastern states had a lower estimated lithium exposure compared with western states (Figure 1). To eliminate potential confounding arising from geospatial variations in lithium exposure, we stratified the study population into 2 regions, west and east, according to geographic conventions, as presented in Figure 1. These geographic regions roughly represent the arid and semiarid west and the humid east, which affect, in part, the occurrence of lithium in groundwater. We reran the regression models using region-specific quintiles of lithium exposure for overall cancer, the entire and sex-stratified populations, female breast cancer, and male prostate cancer. To mitigate exposure misclassification arising from participants’ moving residence, we additionally ran the same regression analyses only among long-term residents (ie, participants who reported living at their current address for at least 3 years).

We conducted several sensitivity analyses to examine the robustness of our results. First, population density appeared to be a potential confounder given the uneven distribution of lithium exposure and cancer incidence across the US. Therefore, we stratified the study population into 3 groups based on the tertiles of population density of the geographic units in this study and ran the regression model in each population density group using group-specific lithium exposure tertiles. Second, we used the lithium concentration groups in groundwater published by the USGS in January 2024 as the exposure (eFigure 2 and eMethods 3 in Supplement 1).^31^ Third, we excluded participants with a history of lithium medication use. Fourth, to avoid potential assumption violations of the Cox proportional hazards regression model, we used Poisson regression to estimate the cancer risks in the full population (eMethods 4 in Supplement 1). Fifth, we assessed the lithium exposure using concentration data from lithium samples collected between 2009 and 2018 and used this new exposure in the regression model. Sixth, we identified all cancer records, including cancer history before enrollment and new cancer cases after enrollment, from both EHRs and cancer history questionnaires and used logistic regression to estimate the odds ratio (OR) for cancer risk (eMethods 4 in Supplement 1). Seventh, because all USGS lithium concentration measurements were from groundwater, we estimated the proportion of the population served by a groundwater source in each 3-digit zip code area and reran our regression analysis in areas with more than 30% of the population served by a groundwater source (eMethods 5 in Supplement 1). Eighth, as arsenic is the most frequently detected trace metal contaminant in groundwater used for drinking water and is a known carcinogen,^32^ we restricted our analyses to areas where the probability of arsenic concentration over 10 μg/L, the EPA standard, was lower than 5% based on the USGS model.^33^ Two-sided *P* < .05 was considered statistically significant. Data were analyzed from September 2023 through October 2024 using the survival package in R, version 4.4.0 (R Project for Statistical Computing).

## Results

### Population Characteristics and Lithium Exposure

A total of 252 178 individuals without a history of cancer were included, with a median follow-up time of 3.6 years (IQR, 3.0-4.3 years) (Table 1). Of the study population, 60.1% were female, 37.9% were male, and 0.9% were other. A total of 21.6% were Black; 20.5%, Hispanic or Latino; 48.8%, White; and 9.1%, other race and ethnicity. Overall, 54.6% were older than 50 years at enrollment (median age, 52 years [IQR, 36-64 years]). Across the lithium exposure quintiles, we observed no substantial difference in the selected characteristics except for race and ethnicity, as the first and fifth quintiles consisted of 31.7% and 31.0% Hispanic or Latino people, respectively, higher than in other quintiles. Notably, the Deprivation Index did not vary across lithium quintiles.

**Table 1.** Selected Characteristics of the US Study Population From the All of Us Research Program^a^

Characteristic	Overall (N = 252 178)	Lithium exposure quintile^b^				
		1 (n = 52 223)	2 (n = 49 235)	3 (n = 50 703)	4 (n = 50 955)	5 (n = 49 062)
Sex assigned at birth						
Female	151 584 (60.1)	32 069 (61.4)	29 975 (60.9)	30 430 (60.0)	30 459 (59.8)	28 651 (58.4)
Male	95 498 (37.9)	19 218 (36.8)	18 150 (36.9)	19 075 (37.6)	19 500 (38.3)	19 555 (39.9)
Other	2395 (0.9)	435 (0.8)	511 (1.0)	543 (1.1)	512 (1.0)	394 (0.8)
Missing	2701 (1.1)	501 (1.0)	599 (1.2)	655 (1.3)	484 (0.9)	462 (0.9)
Household income, $						
<35 000	88 708 (35.2)	17 055 (32.7)	17 838 (36.2)	20 982 (41.4)	13 774 (27.0)	19 059 (38.8)
35 000-49 900	19 419 (7.7)	3422 (6.6)	3880 (7.9)	4106 (8.1)	3408 (6.7)	4603 (9.4)
50 000-74 900	24 532 (9.7)	4239 (8.1)	5148 (10.5)	5221 (10.3)	5041 (9.9)	4883 (10.0)
75 000-149 900	40 322 (16.0)	7254 (13.9)	8314 (16.9)	8198 (16.2)	10 003 (19.6)	6553 (13.4)
≥150 000	23 733 (9.4)	6074 (11.6)	4490 (9.1)	4094 (8.1)	6888 (13.5)	2187 (4.5)
Missing	55 464 (22.0)	14 179 (27.2)	9565 (19.4)	8102 (16.0)	11 841 (23.2)	11 777 (24.0)
Race and ethnicity						
Hispanic or Latino	51 575 (20.5)	16 572 (31.7)	4906 (10.0)	4139 (8.2)	10 736 (21.1)	15 222 (31.0)
Non-Hispanic Black	54 535 (21.6)	9775 (18.7)	14 701 (29.9)	16 945 (33.4)	7999 (15.7)	5115 (10.4)
Non-Hispanic White	123 010 (48.8)	20 796 (39.8)	25 626 (52.0)	25 366 (50.0)	26 113 (51.2)	25 109 (51.2)
Other^c^	23 058 (9.1)	5080 (9.7)	4002 (8.1)	4253 (8.4)	6107 (12.0)	3616 (7.4)
Age at enrollment, y						
18-20	2265 (0.9)	379 (0.7)	346 (0.7)	374 (0.7)	414 (0.8)	752 (1.5)
21-30	33 080 (13.1)	7022 (13.4)	5568 (11.3)	6188 (12.2)	5626 (11.0)	8676 (17.7)
31-40	40 391 (16.0)	9104 (17.4)	7652 (15.5)	7545 (14.9)	7494 (14.7)	8596 (17.5)
41-50	38 638 (15.3)	7868 (15.1)	7711 (15.7)	7581 (15.0)	7740 (15.2)	7738 (15.8)
51-60	51 940 (20.6)	10 774 (20.6)	10 758 (21.9)	11 242 (22.2)	10 323 (20.3)	8843 (18.0)
61-70	50 263 (19.9)	10 007 (19.2)	10 438 (21.2)	10 944 (21.6)	10 838 (21.3)	8036 (16.4)
71-80	28 169 (11.2)	5538 (10.6)	5453 (11.1)	5389 (10.6)	6798 (13.3)	4991 (10.2)
>80	7432 (2.9)	1531 (2.9)	1309 (2.7)	1440 (2.8)	1722 (3.4)	1430 (2.9)
Educational level						
<High school	26 807 (10.6)	6321 (12.1)	4360 (8.9)	4645 (9.2)	5657 (11.1)	5824 (11.9)
High school or equivalent	53 206 (21.1)	10 668 (20.4)	10 875 (22.1)	10 497 (20.7)	8269 (16.2)	12 897 (26.3)
Some college	64 809 (25.7)	11 357 (21.7)	12 365 (25.1)	12 852 (25.3)	12 391 (24.3)	15 844 (32.3)
Undergraduate degree	53 075 (21.0)	11 384 (21.8)	10 683 (21.7)	10 851 (21.4)	12 165 (23.9)	7992 (16.3)
Graduate degree	45 970 (18.2)	10 722 (20.5)	9087 (18.5)	9836 (19.4)	11 170 (21.9)	5155 (10.5)
Missing	8311 (3.3)	1771 (3.4)	1865 (3.8)	2022 (4.0)	1303 (2.6)	1350 (2.8)
Body mass index^d^						
Underweight	3443 (1.4)	750 (1.4)	560 (1.1)	770 (1.5)	702 (1.4)	661 (1.3)
Normal	63 390 (25.1)	14 293 (27.4)	12 008 (24.4)	13 499 (26.6)	13 579 (26.6)	10 011 (20.4)
Overweight	72 279 (28.7)	16 195 (31.0)	13 857 (28.1)	14 315 (28.2)	15 412 (30.2)	12 500 (25.5)
Obesity	101 471 (40.2)	19 912 (38.1)	21 073 (42.8)	20 959 (41.3)	19 353 (38.0)	20 174 (41.1)
Missing	11 595 (4.6)	1073 (2.1)	1737 (3.5)	1160 (2.3)	1909 (3.7)	5716 (11.7)
Ever smoking status						
No	143 604 (56.9)	31 108 (59.6)	27 779 (56.4)	27 936 (55.1)	29 716 (58.3)	27 065 (55.2)
Yes	106 258 (42.1)	20 665 (39.6)	20 793 (42.2)	22 285 (44.0)	20 744 (40.7)	21 771 (44.4)
Missing	2316 (0.9)	450 (0.9)	663 (1.3)	482 (1.0)	495 (1.0)	226 (0.5)
Alcohol drinking status						
No	28 543 (11.3)	7153 (13.7)	5568 (11.3)	4764 (9.4)	5328 (10.5)	5730 (11.7)
Yes	217 727 (86.3)	43 954 (84.2)	42 299 (85.9)	44 518 (87.8)	44 613 (87.6)	42 343 (86.3)
Missing	5908 (2.3)	1116 (2.1)	1368 (2.8)	1421 (2.8)	1014 (2.0)	989 (2.0)
Deprivation Index, median (IQR)	0.33 (0.29-0.39)	0.31 (0.30-0.37)	0.33 (0.29-0.35)	0.33 (0.29-0.39)	0.30 (0.27-0.36)	0.34 (0.33-0.38)

^a^ Data are presented as number (percentage) of participants unless otherwise indicated.

^b^ Quintile 1, 1.3 to 3.6 μg/L; quintile 2, 3.7 to 6.1 μg/L; quintile 3, 6.2 to 7.2 μg/L; quintile 4, 7.3 to 25.5 μg/L; quintile 5, 25.6 to 149.9 μg/L.

^c^ American Indian, Asian, multiracial, or other than those listed.

^d^ Calculated as weight in kilograms divided by height in meters squared. Less than 18.5 indicates underweight; 18.5-24.9, normal; 25.0 to 29.9, overweight; and 30.0 or higher, obesity.

In the overall study population, the median lithium exposure level was 7.0 μg/L (IQR, 4.1-17.8 μg/L) (Figure 1 and eTable 1 in Supplement 1). Participants in the western states had a higher exposure level compared with their counterparts in the eastern states (median, 43.2 μg/L [IQR, 18.3-53.6 μg/L] vs 5.7 μg/L [IQR, 3.6-7.1 μg/L]). During the study period, we identified 7573 incident cancer cases from EHRs, including cases in 4296 of the 151 584 females (2.8%) and 3134 of the 95 498 males (3.3%). The western states had a lower cancer rate compared with the eastern states (1.8% vs 3.5%).

### Full Population Outcomes

In this study, higher lithium exposure was associated with decreased cancer risk, including all cancer types combined, across both females and males and in both the overall population and the population restricted to long-term residents (Table 2). In the overall population, compared with the first quintile of estimated lithium exposure, all higher quintiles were associated with a decreased risk for all cancers combined. For instance, the HR was 0.63 (95% CI, 0.41-0.97) for the third quintile, 0.49 (95% CI, 0.31-0.78) for the fourth quintile, and 0.29 (95% CI, 0.15-0.55) for the fifth quintile. When restricted to long-term residents, there was no association in the second and third quintiles; however, decreased risks were still observed for the fourth (HR, 0.53; 95% CI, 0.33-0.84) and fifth (HR, 0.25; 95% CI, 0.12-0.53) quintiles.

**Table 2.** AHRs and 95% CIs for Cancer Risk in the US Study Population According to Estimated Lithium Exposure From Drinking Groundwater

Lithium exposure quintile^a^	Full population			Long-term residents^b^		
	Participants, No.	Cancer cases, No.^c^	AHR (95% CI)^d^	Participants, No.	Cancer cases, No.^c^	AHR (95% CI)^d^
**All cancers**						
Total						
1	52 223	2390	1 [Reference]	34 138	1917	1 [Reference]
2	49 235	1497	0.43 (0.22-0.85)	31 343	1178	0.65 (0.37-1.14)
3	50 703	1871	0.63 (0.41-0.97)	31 899	1453	0.74 (0.50-1.09)
4	50 955	1510	0.49 (0.31-0.78)	30 164	985	0.53 (0.33-0.84)
5	49 062	301	0.29 (0.15-0.55)	30 345	432	0.25 (0.12-0.53)
Females						
1	32 069	1308	1 [Reference]	21 912	1056	1 [Reference]
2	29 975	861	0.68 (0.38-1.22)	17 475	571	0.62 (0.33-1.16)
3	30 430	1071	0.75 (0.48-1.16)	20 374	849	0.79 (0.52-1.20)
4	28 799	855	0.65 (0.41-1.04)	18 904	618	0.60 (0.37-0.98)
5	30 311	201	0.17 (0.07-0.42)	19 576	302	0.32 (0.15-0.66)
Males						
1	19 218	1044	1 [Reference]	11 907	842	1 [Reference]
2	19 183	616	0.61 (0.37-1.02)	11 314	483	0.61 (0.35-1.05)
3	19 151	809	0.96 (0.68-1.35)	11 639	604	0.74 (0.51-1.08)
4	19 064	547	0.46 (0.30-0.71)	11 508	388	0.45 (0.27-0.73)
5	18 882	118	0.13 (0.04-0.38)	11 573	187	0.24 (0.11-0.52)
**Breast cancer, only female**						
1	31 100	426	1 [Reference]	32 481	355	1 [Reference]
2	29 330	280	0.65 (0.36-1.18)	30 328	227	0.65 (0.36-1.18)
3	29 624	353	0.76 (0.48-1.19)	30 648	290	0.77 (0.50-1.20)
4	29 895	353	0.76 (0.45-1.26)	29 390	234	0.68 (0.40-1.16)
5	28 536	60	0.16 (0.06-0.41)	29 998	98	0.32 (0.14-0.70)
**Prostate cancer, only male**						
1	18 655	313	1 [Reference]	32 389	263	1 [Reference]
2	18 684	156	0.50 (0.29-0.87)	30 250	149	0.59 (0.34-1.05)
3	19 058	262	0.98 (0.70-1.38)	30 556	198	0.76 (0.51-1.14)
4	18 167	133	0.36 (0.22-0.58)	29 263	107	0.39 (0.21-0.71)
5	18 593	33	0.15 (0.04-0.51)	29 956	56	0.24 (0.11-0.53)
**Bladder and urinary cancer**						
1	49 787	73	1 [Reference]	32 192	66	1 [Reference]
2	48 057	41	0.53 (0.26-1.08)	30 134	33	0.49 (0.23-1.03)
3	49 326	58	0.73 (0.42-1.26)	30 406	48	0.69 (0.39-1.22)
4	48 491	22	0.24 (0.14-0.41)	29 170	<20	0.19 (0.10-0.37)
5	48 759	<20	0.11 (0.04-0.35)	29 908	<20	0.12 (0.05-0.30)
**CNS cancer**						
1	49 752	38	1 [Reference]	32 151	25	1 [Reference]
2	48 041	25	0.67 (0.35-1.30)	30 116	<20	0.65 (0.25-1.68)
3	49 296	28	0.77 (0.48-1.23)	30 377	<20	0.82 (0.42-1.62)
4	48 501	32	0.81 (0.46-1.41)	29 175	<20	0.75 (0.37-1.54)
5	48 756	<20	0.10 (0.02-0.42)	29 907	<20	0.27 (0.08-0.92)
**Colorectal cancer**						
1	49 800	86	1 [Reference]	32 193	67	1 [Reference]
2	48 082	66	0.86 (0.46-1.60)	30 150	49	0.79 (0.41-1.52)
3	49 348	80	0.95 (0.60-1.51)	30 421	63	0.95 (0.59-1.53)
4	48 521	52	0.60 (0.34-1.05)	29 189	33	0.51 (0.28-0.92)
5	48 765	<20	0.18 (0.06-0.57)	29 914	<20	0.23 (0.10-0.53)
**Kidney cancer**						
1	49 770	56	1 [Reference]	32 167	41	1 [Reference]
2	48 060	44	0.72 (0.37-1.42)	30 132	31	0.72 (0.37-1.38)
3	49 325	57	0.85 (0.50-1.47)	30 396	38	0.79 (0.46-1.37)
4	48 505	36	0.52 (0.29-0.95)	29 180	24	0.51 (0.27-0.97)
5	48 760	<20	0.14 (0.04-0.52)	29 912	<20	0.28 (0.11-0.71)
**Leukemia**						
1	49 769	55	1 [Reference]	32 170	44	1 [Reference]
2	48 054	38	0.72 (0.39-1.32)	30 129	28	0.63 (0.32-1.26)
3	49 316	48	0.89 (0.51-1.55)	30 397	39	0.93 (0.51-1.69)
4	48 534	65	1.16 (0.67-2.01)	29 201	45	1.06 (0.59-1.90)
5	48 766	<20	0.22 (0.07-0.68)	29 920	20	0.40 (0.15-1.04)
**NHL**						
1	49 819	105	1 [Reference]	32 206	80	1 [Reference]
2	48 077	61	0.65 (0.35-1.21)	30 148	47	0.65 (0.35-1.21)
3	49 344	76	0.75 (0.42-1.33)	30 422	64	0.85 (0.51-1.41)
4	48 534	65	0.63 (0.36-1.10)	29 193	37	0.52 (0.29-0.92)
5	48 765	<20	0.14 (0.04-0.48)	29 927	27	0.36 (0.15-0.90)
**Thyroid cancer**						
1	49 849	135	1 [Reference]	32 242	116	1 [Reference]
2	48 096	80	0.74 (0.32-1.69)	30 161	60	0.63 (0.28-1.43)
3	49 350	82	0.72 (0.35-1.46)	30 411	53	0.54 (0.25-1.17)
4	48 519	50	0.41 (0.23-0.73)	29 191	35	0.36 (0.18-0.71)
5	48 761	<20	0.10 (0.03-0.32)	29 912	<20	0.14 (0.05-0.38)

Abbreviations: AHR, adjusted hazard ratio; CNS, central nervous system; NHL, non-Hodgkin lymphoma.

^a^ Quintile 1, 1.3 to 3.6 μg/L; quintile 2, 3.7 to 6.1 μg/L; quintile 3, 6.2 to 7.2 μg/L; quintile 4, 7.3 to 25.5 μg/L; quintile 5, 25.6 to 149.9 μg/L.

^b^ Refers to participants who reported living at their current address for at least 3 years.

^c^ Per the All of Us Research Program privacy policy, data for cells with frequency less than 20 are not reported.

^d^ Three stratified terms were included: sex at birth, race and ethnicity, and age. Adjusted for educational level, household income, smoking status, alcohol drinking status, and the deprivation index of the residential address.

The association of the highest exposure quintile with lower risk of all cancers combined persisted when the population was stratified into males and females. Specifically, the HR for the fifth quintile was 0.17 (95% CI, 0.07-0.42) in females and 0.13 (95% CI, 0.04-0.38) in males. When restricted to long-term residents, the HRs for the fifth quintile were attenuated (HR, 0.32 [95% CI, 0.15-0.66] in females and 0.24 [95% CI, 0.11-0.52] in males).

Inverse associations were also observed for all individual cancer types (breast, prostate, bladder and urinary, CNS, kidney, colorectal, leukemia, NHL, and thyroid cancer) investigated in this study. The fifth quintile of exposure was associated with the lowest risks, with HRs ranging from 0.10 (95% CI, 0.02-0.42) for CNS cancer and 0.10 (95% CI, 0.03-0.32) for thyroid cancer to 0.22 (95% CI, 0.07-0.68) for leukemia, and lithium exposure in the fifth quintile was associated with lower risk of all cancer types. The associations remained when analysis was restricted to long-term residents, except for the risk of leukemia, for which there was no association (HR, 0.40; 95% CI, 0.15-1.04) for the fifth quintile of lithium exposure.

**Figure 2.**
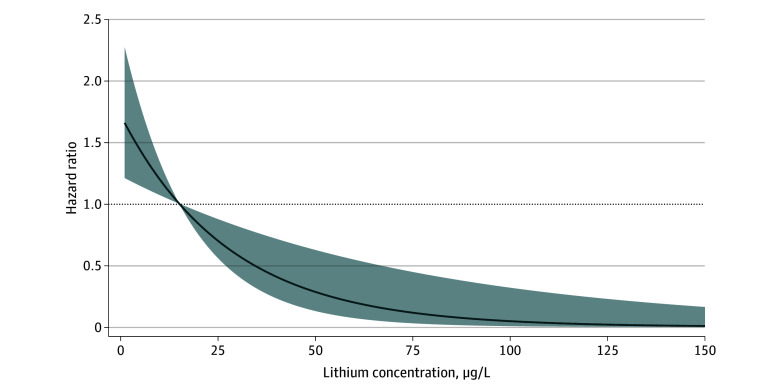
Nonlinear Association Between Estimated Lithium Exposure From Drinking Groundwater and Risk of All Cancers Shading indicates 95% CIs.

Nonlinear analysis corroborated our main findings. An L-shaped relationship was observed between lithium exposure and risk for all cancers (Figure 2), with the curve flattening with higher concentrations. The nonlinear curves for other outcomes can be found in eFigure 3 in Supplement 1.

### Region-Stratified Analysis

Similar inverse associations were observed in both western and eastern states. Lower cancer risks were observed in the western states, where the estimated lithium exposure level was higher. Specifically, the HR for all cancers was 0.08 (95% CI, 0.01-0.57) in the western states vs 0.64 (95% CI, 0.46-0.90) in the eastern states for the fourth quintile and 0.01 (95% CI, 0.00-0.09) in the west vs 0.34 (95% CI, 0.21-0.57) in the east for the fifth quintile (Table 3). Results from long-term residents in the region-stratified analysis were consistent with those in the full population (eTable 2 in Supplement 1). Results from all sensitivity analyses that stratified study population by population density (eTable 3 in Supplement 1), used USGS lithium concentration group (eAppendix and eTable 4 in Supplement 1), excluded participants with lithium medication history (eTable 5 in Supplement 1), used Poisson regression (eTables 6 and 7 in Supplement 1), assessed lithium exposure based on concentration data between 2009 and 2018 (eTables 8 and 9 in Supplement 1), included all cancer records at any time from EHRs and questionnaires (eTables 10 and 11 in Supplement 1), only included areas where more than 30% of the population used groundwater as a drinking water supply (eTable 12 in Supplement 1), and only included areas with low arsenic concentration (eTable 13 in Supplement 1) were also consistent with our main results.

**Table 3.** AHRs and 95% CIs for Cancer Risk According to Estimated Lithium Exposure From Drinking Groundwater in the US Study Population Stratified by Geographic Regions

Lithium exposure quintile	Western states				Eastern states			
	Lithium level, median (range), μg/L	Participants, No.	Cancer cases, No.^a^	AHR (95% CI)^b^	Lithium level, median (range), μg/L	Participants, No.	Cancer cases, No.^a^	AHR (95% CI)^b^
**All cancers**								
Total								
1	15.7 (2.4-17.8)	19 214	724	1 [Reference]	2.3 (1.3-3.4)	34 843	1948	1 [Reference]
2	20.2 (17.9-29.6)	12 535	452	1.06 (0.50-2.23)	3.9 (3.5-4.4)	37 202	1011	0.44 (0.25-0.79)
3	43.1 (29.7-44.0)	15 966	204	0.32 (0.11-1.00)	5.9 (4.5-6.6)	31 875	912	0.61 (0.36-1.05)
4	53.6 (44.1-65.8)	21 117	63	0.08 (0.01-0.57)	7.0 (6.7-7.2)	42 021	1623	0.64 (0.46-0.90)
5	70.0 (65.9-149.9)	10 343	<20	0.01 (0.00-0.09)	7.8 (7.3-68.2)	27 062	629	0.34 (0.21-0.57)
Females								
1	15.7 (2.4-17.8)	18 892	442	1 [Reference]	2.3 (1.3-3.4)	21 372	1052	1 [Reference]
2	20.2 (17.9-29.6)	13 682	235	0.56 (0.33-0.93)	3.9 (3.5-4.4)	20 724	574	0.48 (0.26-0.89)
3	43.1 (29.7-44.0)	14 893	120	0.74 (0.42-1.28)	5.9 (4.5-6.6)	20 925	526	0.56 (0.31-1.03)
4	53.6 (44.1-65.8)	19 348	36	0.56 (0.38-0.83)	7.0 (6.7-7.2)	25 547	932	0.65 (0.45-0.96)
5	70.0 (65.9-149.9)	11 731	<20	0.33 (0.19-0.59)	7.8 (7.3-68.2)	16 251	376	0.37 (0.23-0.62)
Males								
1	15.7 (2.4-17.8)	7247	272	1 [Reference]	2.3 (1.3-3.4)	12 951	858	1 [Reference]
2	20.2 (17.9-29.6)	5208	215	1.33 (0.79-2.23)	3.9 (3.5-4.4)	13 434	429	0.44 (0.26-0.75)
3	43.1 (29.7-44.0)	9824	80	0.23 (0.06-0.90)	5.9 (4.5-6.6)	12 317	364	0.50 (0.28-0.88)
4	53.6 (44.1-65.8)	4470	27	0.14 (0.01-1.33)	7.0 (6.7-7.2)	15 564	650	0.86 (0.60-1.21)
5	70.0 (65.9-149.9)	4278	0	NA	7.8 (7.3-68.2)	10 205	239	0.30 (0.17-0.51)
**Breast cancer, only female**								
1	15.7 (2.4-17.8)	11 387	173	1 [Reference]	2.3 (1.3-3.4)	20 583	345	1 [Reference]
2	20.2 (17.9-29.6)	7137	121	1.53 (0.77-3.06)	3.9 (3.5-4.4)	22 526	183	0.43 (0.23-0.79)
3	43.1 (29.7-44.0)	9290	43	0.33 (0.12-0.97)	5.9 (4.5-6.6)	18 312	175	0.67 (0.36-1.22)
4	53.6 (44.1-65.8)	12 532	<20	0.06 (0.01-0.38)	7.0 (6.7-7.2)	24 843	303	0.65 (0.44-0.96)
5	70.0 (65.9-149.9)	5903	<20	0.04 (0.00-0.49)	7.8 (7.3-68.2)	15 972	116	0.35 (0.20-0.64)
**Prostate cancer, only male**								
1	15.7 (2.4-17.8)	7027	55	1 [Reference]	2.3 (1.3-3.4)	12 538	260	1 [Reference]
2	20.2 (17.9-29.6)	5740	67	1.48 (0.70-3.09)	3.9 (3.5-4.4)	12 894	112	0.38 (0.23-0.63)
3	43.1 (29.7-44.0)	9065	<20	0.22 (0.04-1.32)	5.9 (4.5-6.6)	12 534	114	0.48 (0.26-0.87)
4	53.6 (44.1-65.8)	4449	<20	0.17 (0.01-1.90)	7.0 (6.7-7.2)	14 600	198	0.92 (0.66-1.28)
5	70.0 (65.9-149.9)	4278	0	NA	7.8 (7.3-68.2)	10 032	74	0.29 (0.16-0.51)

Abbreviations: AHR, adjusted hazard ratio; NA, not applicable.

^a^ Per the All of Us Research Program privacy policy, data for cells with frequency less than 20 are not reported.

^b^ Three stratified terms were included: sex at birth, race and ethnicity, and age. Adjusted for educational level, household income, smoking status, alcohol drinking status, and the deprivation index of the residential address.

## Discussion

In this nationwide cohort study, we observed that higher estimated lithium exposure in drinking groundwater was consistently associated with lower cancer risk. The associations were observed for both males and females and for all major cancer types. Moreover, despite the substantial disparities in lithium concentration in groundwater between the 2 US regions, the associations with lower cancer risk persisted in both eastern and western states, with associations more pronounced in the western states, where the lithium exposure was higher. These findings were unexpected because lithium is considered to be a contaminant in drinking water.^26,34^ However, these findings were also consistent with emerging evidence of lithium’s anticancer effects in both observational and experimental studies.^10,11,12,35,36,37^

One notable disparity in this study was the regional difference in lithium exposure level and cancer rates between the western and eastern states. We observed a higher lithium exposure level and a lower cancer rate in the western states. Other than lithium concentrations in drinking water, other factors may contribute to this regional disparity. For instance, climate variables such as temperature could play a role, as studies have suggested potential links between temperature and human health.^38^ Other environmental factors, including air quality and UV radiation exposure, may also influence regional cancer rates.^39^ Future analyses could incorporate these variables to better understand their potential contributions. However, due to the focus and scope of this study, these factors were not included.

The associations between lithium exposure and reduced cancer risk observed in this study do not necessarily promote intake of lithium-rich water as a public health strategy. Clinical evidence has illustrated an adverse association between lithium medication and kidney and thyroid functions.^9^ However, the lithium-related toxic effects appeared to only be related to higher lithium doses. For example, a clinical trial reported no significant changes in kidney function among patients randomized to low-dose lithium treatment compared with the placebo group.^40^ The serum lithium levels were maintained at 0.25 to 0.50 mEq/L (to convert to mmol/L, multiply by 1) among the treatment group in that study,^40^ which would typically take about 40 to 150 mg/d of elemental lithium to achieve. A more recent, prospective cohort study did not find any increased risk for kidney disease in lithium users except for those with high serum levels (>1.0 mEq/L).^41^ Considering the average daily water consumption is about 1.3 L for adults, even those living in areas with the highest lithium concentration in the drinking water would only ingest about 0.2 mg of lithium each day from water intake. This daily dose is highly unlikely to cause kidney disorders based on current evidence given the substantially lower environmental lithium exposure level.

### Strengths and Limitations

A strength of this study is that it was a large, nationwide, diverse longitudinal study. The comprehensive EHR data allowed us to retrieve key variables. The lithium data used in this study included 4700 monitoring sites distributed across the contiguous US, standing out as the largest data sample on lithium measures in groundwater in the literature to our knowledge. The diverse populations in All of Us make the results more generalizable compared with a prior study restricted to certain regions or racial, ethnic, and socioeconomic groups.^28^

Several limitations should be considered. First, exposure misclassification cannot be ruled out. Kriging is a geospatial method widely used in prior studies on lithium exposure.^16,23^ However, this method is data driven and cannot accurately reflect the actual exposure level that can be influenced by subtle geographic variations, meteorologic factors, and anthropogenic activities. The 3-digit zip code level in the All of Us Research Program limited a finer exposure assessment, leading to potential misclassification. This limitation precludes a more sophisticated dose-response analysis about lithium exposure. Second, we could not obtain data on cancer stage, positive lymph nodes, and other cancer characteristics in the All of Us Research Program, preventing investigations of the underlying mechanisms in the lithium exposures. Third, we did not have information about bottled water consumption in the study population. Statistics indicated an increasing trend of bottled water consumption in the US,^42^ with roughly 10% of annual water intake for each adult, which may raise concerns about the accuracy of the exposure assessment. Fourth, while we controlled for key variables and conducted sensitivity analyses, data limitations prevented adjusting for other groundwater contaminants potentially correlated with lithium, which may influence cancer risk estimates.

## Conclusions

In this cohort study of 252 178 participants, we found that higher lithium exposure in drinking groundwater was associated with reduced cancer risk in the US general population both for cancer overall and for specific cancer types. Since the potential biological mechanisms underlying such protective effects remain unclear, studies on the full range of physical health effects and outcomes in relation to environmental lithium exposure are warranted.

## Notes

Supplement 1. **eMethods 1.** Adjusted Covariates **eMethods 2.** Details About Kriging for Lithium Exposure Assessment in the Contiguous US **eMethods 3.** US Geological Survey Model for Groundwater Lithium Concentration Groups **eMethods 4.** Poisson Regression and Logistic Regression Models in Sensitivity Analyses **eMethods 5.** Estimated Proportion of Population Covered by Groundwater Supply at the 3-Digit Zip Code Level **eAppendix.** Results Using USGS Lithium Concentration Classification Based on Machine Learning Model **eFigure 1.** Spatial Distribution of Wells for Lithium Concentration Measure and Final Kriging Map of Estimated Lithium Concentration in the Contiguous US **eFigure 2.** USGS Lithium Concentration Groups for Public Supply of Drinking Groundwater at the 3-Digit Zip Code Level in the Contiguous US **eFigure 3.** Nonlinear Association Between Lithium Exposure Level and Cancer Outcomes **eTable 1.** Distribution of Estimated Lithium Exposure From Drinking Groundwater in the US Study Population, Categorized Into Quintiles **eTable 2.** Hazard Ratios and 95% CIs for Cancer Risk According to Estimated Lithium Exposure From Drinking Groundwater, Stratified By Geographic Regions Among Long-Term Residents **eTable 3.** Hazard Ratios and 95% CIs for Cancer Risk in the US Study Population, Stratified by Population Density **eTable 4.** Results Based on US Geological Survey Lithium Concentration Group Model for the US Study Population **eTable 5.** Hazard Ratios and 95% CIs for Cancer Risk in the US Study Population Without Lithium Medication History **eTable 6.** Incidence Rate Ratios and 95% CIs From Poisson Regression for Cancer Risk in the US Study Population According to Estimated Lithium Exposure From Drinking Groundwater **eTable 7.** Incidence Rate Ratios and 95% CIs From Poisson Regression for Cancer Risk According to Estimated Lithium Exposure From Drinking Groundwater in the US Study Population, Stratified by Geographic Regions **eTable 8.** Hazard Ratios and 95% CIs for Cancer Risk in the US Study Population According to Estimated Lithium Exposure From Drinking Groundwater, Based on 2009-2018 Lithium Measure Data **eTable 9.** Hazard Ratios and 95% CIs for Cancer Risk in the US Study Population According to Estimated Lithium Exposure From Drinking Groundwater, Based on 2009-2018 Lithium Measure Data and Stratified by Geographic Regions **eTable 10.** Odds Ratios and 95% CIs for Cancer Record at Any Time in the US Study Population From Both Electronic Health Records and Questionnaires, According to Estimated Lithium Exposure From Drinking Groundwater **eTable 11.** Odds Ratios and 95% CIs for Cancer Record at Any Time in the US Study Population From Both Electronic Health Records and Questionnaires, According to Estimated Lithium Exposure From Drinking Groundwater Stratified by Western and Eastern States **eTable 12.** Hazard Ratios and 95% CIs for Cancer Risk in the US Study Population According to Estimated Lithium Exposure in Areas Where More Than 30% of the Population Uses Drinking Water Supplied by Groundwater **eTable 13.** Hazard Ratios and 95% CIs for Cancer Risk in the US Study Population According to Estimated Lithium Exposure From Drinking Groundwater in Areas With Low Arsenic Concentration

Supplement 2.
Data Sharing Statement

## References

[1] Lindsey BD, Belitz K, Cravotta CA III, Toccalino PL, Dubrovsky NM. Lithium in groundwater used for drinking-water supply in the United States. Sci Total Environ. 2021;767:144691. doi:10.1016/j.scitotenv.2020.144691 33454610

[2] Memon A, Rogers I, Fitzsimmons SMDD, . Association between naturally occurring lithium in drinking water and suicide rates: systematic review and meta-analysis of ecological studies. Br J Psychiatry. 2020;217(6):667-678. doi:10.1192/bjp.2020.128 32716281

[3] Gelenberg AJ, Kane JM, Keller MB, . Comparison of standard and low serum levels of lithium for maintenance treatment of bipolar disorder. N Engl J Med. 1989;321(22):1489-1493. doi:10.1056/NEJM198911303212201 2811970

[4] Yatham LN, Kennedy SH, Parikh SV, . Canadian Network for Mood and Anxiety Treatments (CANMAT) and International Society for Bipolar Disorders (ISBD) 2018 guidelines for the management of patients with bipolar disorder. Bipolar Disord. 2018;20(2):97-170. doi:10.1111/bdi.12609 29536616 PMC5947163

[5] Malhi GS, Gessler D, Outhred T. The use of lithium for the treatment of bipolar disorder: recommendations from clinical practice guidelines. J Affect Disord. 2017;217:266-280. doi:10.1016/j.jad.2017.03.052 28437764

[6] Swann AC, Bowden CL, Morris D, . Depression during mania: treatment response to lithium or divalproex. Arch Gen Psychiatry. 1997;54(1):37-42. doi:10.1001/archpsyc.1997.01830130041008 9006398

[7] Eskalith. Prescribing information. GlaxoSmithKline; 2003. Accessed March 4, 2024. https://www.accessdata.fda.gov/drugsatfda_docs/label/2004/16860slr074,18152slr020_eskalith_lbl.pdf

[8] Bocchetta A, Loviselli A. Lithium treatment and thyroid abnormalities. Clin Pract Epidemiol Ment Health. 2006;2:23. doi:10.1186/1745-0179-2-23 16968542 PMC1584230

[9] Shine B, McKnight RF, Leaver L, Geddes JR. Long-term effects of lithium on renal, thyroid, and parathyroid function: a retrospective analysis of laboratory data. Lancet. 2015;386(9992):461-468. doi:10.1016/S0140-6736(14)61842-0 26003379

[10] Huang RY, Hsieh KP, Huang WW, Yang YH. Use of lithium and cancer risk in patients with bipolar disorder: population-based cohort study. Br J Psychiatry. 2016;209(5):393-399. doi:10.1192/bjp.bp.116.181362 27388574

[11] Anmella G, Fico G, Lotfaliany M, . Risk of cancer in bipolar disorder and the potential role of lithium: international collaborative systematic review and meta-analyses. Neurosci Biobehav Rev. 2021;126:529-541. doi:10.1016/j.neubiorev.2021.03.034 33831461

[12] Asgari MM, Chien AJ, Tsai AL, Fireman B, Quesenberry CP Jr. Association between lithium use and melanoma risk and mortality: a population-based study. J Invest Dermatol. 2017;137(10):2087-2091. doi:10.1016/j.jid.2017.06.002 28629629

[13] Yang C, Zhu B, Zhan M, Hua ZC. Lithium in cancer therapy: friend or foe? Cancers (Basel). 2023;15(4):1095. doi:10.3390/cancers15041095 36831437 PMC9954674

[14] Brown KM, Tracy DK. Lithium: the pharmacodynamic actions of the amazing ion. Ther Adv Psychopharmacol. 2013;3(3):163-176. doi:10.1177/2045125312471963 24167688 PMC3805456

[15] Berridge MJ, Downes CP, Hanley MR. Neural and developmental actions of lithium: a unifying hypothesis. Cell. 1989;59(3):411-419. doi:10.1016/0092-8674(89)90026-3 2553271

[16] Kessing LV, Gerds TA, Knudsen NN, . Association of lithium in drinking water with the incidence of dementia. JAMA Psychiatry. 2017;74(10):1005-1010. doi:10.1001/jamapsychiatry.2017.2362 28832877 PMC5710473

[17] Fajardo VA, Fajardo VA, LeBlanc PJ, MacPherson REK. Examining the relationship between trace lithium in drinking water and the rising rates of age-adjusted Alzheimer’s disease mortality in Texas. J Alzheimers Dis. 2018;61(1):425-434. doi:10.3233/JAD-170744 29103043 PMC7592673

[18] Palmer A, Cates ME, Gorman G. The association between lithium in drinking water and incidence of suicide across 15 Alabama counties. Crisis. 2019;40(2):93-99. doi:10.1027/0227-5910/a000535 30052075

[19] Izsak B, Hidvegi A, Balint L, . Investigation of the association between lithium levels in drinking water and suicide mortality in Hungary. J Affect Disord. 2022;298(Pt A):540-547. doi:10.1016/j.jad.2021.11.041 34800573

[20] Shimodera S, Koike S, Ando S, . Lithium levels in tap water and psychotic experiences in a general population of adolescents. Schizophr Res. 2018;201:294-298. doi:10.1016/j.schres.2018.05.019 29895414

[21] Kohno K, Ishii N, Hirakawa H, Terao T. Lithium in drinking water and crime rates in Japan: cross-sectional study. BJPsych Open. 2020;6(6):e122. doi:10.1192/bjo.2020.63 33054891 PMC7576670

[22] Giotakos O, Tsouvelas G, Nisianakis P, . A negative association between lithium in drinking water and the incidences of homicides, in Greece. Biol Trace Elem Res. 2015;164(2):165-168. doi:10.1007/s12011-014-0210-6 25556933

[23] Liew Z, Meng Q, Yan Q, . Association between estimated geocoded residential maternal exposure to lithium in drinking water and risk for autism spectrum disorder in offspring in Denmark. JAMA Pediatr. 2023;177(6):617-624. doi:10.1001/jamapediatrics.2023.0346 37010840 PMC10071398

[24] Schullehner J, Paksarian D, Hansen B, . Lithium in drinking water associated with adverse mental health effects. Schizophr Res. 2019;210:313-315. doi:10.1016/j.schres.2019.06.016 31285074

[25] Harari F, Langeén M, Casimiro E, . Environmental exposure to lithium during pregnancy and fetal size: a longitudinal study in the Argentinean Andes. Environ Int. 2015;77:48-54. doi:10.1016/j.envint.2015.01.011 25645381

[26] US Environmental Protection Agency. Contaminant Candidate List 5—CCL 5. 2024. Accessed March 1, 2024. https://www.epa.gov/ccl/contaminant-candidate-list-5-ccl-5

[27] US Environmental Protection Agency. Technical fact sheet—lithium in drinking water, a resource for primary agencies. EPA 815-F-23-007. 2023. Accessed March 1, 2024. https://www.epa.gov/system/files/documents/2023-11/ucmr5-technical-fact-sheet-lithium-in-drinking-water.pdf

[28] Denny JC, Rutter JL, Goldstein DB, ; All of Us Research Program Investigators. The “All of Us” research program. N Engl J Med. 2019;381(7):668-676. doi:10.1056/NEJMsr1809937 31412182 PMC8291101

[29] Lindsey BD, Belitz K, Cravotta CA III, Toccalino PL, Dubrovsky NM. Inorganic constituent and ancillary data for evaluation of lithium in groundwater in the United States, 1991-2018: US Geological Survey data release. December 28, 2020. Accessed June 29, 2024. https://www.sciencebase.gov/catalog/item/5f5a676c82cefd9f20866c9a

[30] Shah AD, Bartlett JW, Carpenter J, Nicholas O, Hemingway H. Comparison of random forest and parametric imputation models for imputing missing data using MICE: a CALIBER study. Am J Epidemiol. 2014;179(6):764-774. doi:10.1093/aje/kwt312 24589914 PMC3939843

[31] Lombard MA, Brown EE, Saftner DM, . Estimating lithium concentrations in groundwater used as drinking water for the conterminous United States. Environ Sci Technol. 2024;58(2):1255-1264. doi:10.1021/acs.est.3c03315 38164924 PMC10795177

[32] Belitz K, Fram MS, Lindsey BD, . Quality of groundwater used for public supply in the continental United States: a comprehensive assessment. ACS ES&T Water. 2022;2(12):2645-2656. doi:10.1021/acsestwater.2c00390

[33] Lombard MA, Bryan MS, Jones DK, . Machine learning models of arsenic in private wells throughout the conterminous United States as a tool for exposure assessment in human health studies. Environ Sci Technol. 2021;55(8):5012-5023. doi:10.1021/acs.est.0c05239 33729798 PMC8852770

[34] US Geological Survey. Health-based screening levels for evaluating water-quality data. Accessed June 29, 2024. https://www.usgs.gov/tools/health-based-screening-levels-evaluating-water-quality-data

[35] Klein PS, Melton DA. A molecular mechanism for the effect of lithium on development. Proc Natl Acad Sci U S A. 1996;93(16):8455-8459. doi:10.1073/pnas.93.16.8455 8710892 PMC38692

[36] Zhang F, Phiel CJ, Spece L, Gurvich N, Klein PS. Inhibitory phosphorylation of glycogen synthase kinase-3 (GSK-3) in response to lithium: evidence for autoregulation of GSK-3. J Biol Chem. 2003;278(35):33067-33077. doi:10.1074/jbc.M212635200 12796505

[37] O’Brien WT, Klein PS. Validating GSK3 as an in vivo target of lithium action. Biochem Soc Trans. 2009;37(Pt 5):1133-1138. doi:10.1042/BST0371133 19754466 PMC2747042

[38] Radua J, De Prisco M, Oliva V, Fico G, Vieta E, Fusar-Poli P. Impact of air pollution and climate change on mental health outcomes: an umbrella review of global evidence. World Psychiatry. 2024;23(2):244-256. doi:10.1002/wps.21219 38727076 PMC11083864

[39] Reid CE, Brauer M, Johnston FH, Jerrett M, Balmes JR, Elliott CT. Critical review of health impacts of wildfire smoke exposure. Environ Health Perspect. 2016;124(9):1334-1343. doi:10.1289/ehp.1409277 27082891 PMC5010409

[40] Aprahamian I, Santos FS, dos Santos B, . Long-term, low-dose lithium treatment does not impair renal function in the elderly: a 2-year randomized, placebo-controlled trial followed by single-blind extension. J Clin Psychiatry. 2014;75(7):e672-e678. doi:10.4088/JCP.13m08741 25093483

[41] Bosi A, Clase CM, Ceriani L, . Absolute and relative risks of kidney outcomes associated with lithium vs valproate use in Sweden. JAMA Netw Open. 2023;6(7):e2322056. doi:10.1001/jamanetworkopen.2023.22056 37418264 PMC10329212

[42] Ridder M. Per capita consumption of bottled water in the United States from 1999-2023. Statista. Accessed March 24, 2024. https://www.statista.com/statistics/183377/per-capita-consumption-of-bottled-water-in-the-us-since-1999/

