# Errors in Table 3

**DOI:** 10.1001/jamanetworkopen.2025.18037

**Published:** 2025-05-27

In the Original Investigation titled “Cancer Risk and Estimated Lithium Exposure in Drinking Groundwater in the US,”^1^ published February 20, 2025, in Table 3, the numbers of cases for all cancers among females in Western states were incorrect for all lithium levels. This article has been corrected.^1^
