# Supplementary material for: Cancer Risk and Estimated Lithium Exposure in Drinking Groundwater in the US
Source: JAMA Netw Open. 2025 Feb 20;8(2):e2460854. doi: 10.1001/jamanetworkopen.2024.60854 (PMC11843356; doi:10.1001/jamanetworkopen.2024.60854)
Supplement: Supplement 1. — eMethods 1. Adjusted Covariates eMethods 2. Details About Kriging for Lithium Exposure Assessment in the Contiguous US eMethods 3. US Geological Survey Model for Groundwater Lithium Concentration Groups eMethods 4. Poisson Regression and Logistic Regression Models in Sensitivity Analyses eMethods 5. Estimated Proportion of Population Covered by Groundwater Supply at the 3-Digit Zip Code Level eAppendix. Results Using USGS Lithium Concentration Classification Based on Machine Learning Model eFigure 1. Spatial Distribution of Wells for Lithium Concentration Measure and Final Kriging Map of Estimated Lithium Concentration in the Contiguous US eFigure 2. USGS Lithium Concentration Groups for Public Supply of Drinking Groundwater at the 3-Digit Zip Code Level in the Contiguous US eFigure 3. Nonlinear Association Between Lithium Exposure Level and Cancer Outcomes eTable 1. Distribution of Estimated Lithium Exposure From Drinking Groundwater in the US Study Population, Categorized Into Quintiles eTable 2. Hazard Ratios and 95% CIs for Cancer Risk According to Estimated Lithium Exposure From Drinking Groundwater, Stratified By Geographic Regions Among Long-Term Residents eTable 3. Hazard Ratios and 95% CIs for Cancer Risk in the US Study Population, Stratified by Population Density eTable 4. Results Based on US Geological Survey Lithium Concentration Group Model for the US Study Population eTable 5. Hazard Ratios and 95% CIs for Cancer Risk in the US Study Population Without Lithium Medication History eTable 6. Incidence Rate Ratios and 95% CIs From Poisson Regression for Cancer Risk in the US Study Population According to Estimated Lithium Exposure From Drinking Groundwater eTable 7. Incidence Rate Ratios and 95% CIs From Poisson Regression for Cancer Risk According to Estimated Lithium Exposure From Drinking Groundwater in the US Study Population, Stratified by Geographic Regions eTable 8. Hazard Ratios and 95% CIs for Cancer Risk in the US Study Population According to E [file jamanetwopen-e2460854-s001.pdf]

Supplementary Online Content

Luo J, Zheng L, Jin Z, et al. Cancer risk and estimated lithium exposure in drinking groundwater in the US. *JAMA Netw Open*. 2025;8(2):e2460854. doi:10.1001/jamanetworkopen.2024.60854

eMethods 1. Adjusted Covariates

eMethods 2. Details About Kriging for Lithium Exposure Assessment in the Contiguous US

eMethods 3. US Geological Survey Model for Groundwater Lithium Concentration Groups

eMethods 4. Poisson Regression and Logistic Regression Models in Sensitivity Analyses

eMethods 5. Estimated Proportion of Population Covered by Groundwater Supply at the 3-Digit Zip Code Level

eAppendix. Results Using USGS Lithium Concentration Classification Based on Machine Learning Model

eFigure 1. Spatial Distribution of Wells for Lithium Concentration Measure and Final Kriging Map of Estimated Lithium Concentration in the Contiguous US

eFigure 2. USGS Lithium Concentration Groups for Public Supply of Drinking Groundwater at the 3-Digit Zip Code Level in the Contiguous US

eFigure 3. Nonlinear Association Between Lithium Exposure Level and Cancer Outcomes

eTable 1. Distribution of Estimated Lithium Exposure From Drinking Groundwater in the US Study Population, Categorized Into Quintiles

eTable 2. Hazard Ratios and 95% CIs for Cancer Risk According to Estimated Lithium Exposure From Drinking Groundwater, Stratified By Geographic Regions Among Long-Term Residents

eTable 3. Hazard Ratios and 95% CIs for Cancer Risk in the US Study Population, Stratified by Population Density

eTable 4. Results Based on US Geological Survey Lithium Concentration Group Model for the US Study Population

© 2025 Luo J et al. *JAMA Network Open*.

eTable 5. Hazard Ratios and 95% CIs for Cancer Risk in the US Study Population Without Lithium Medication History

eTable 6. Incidence Rate Ratios and 95% CIs From Poisson Regression for Cancer Risk in the US Study Population According to Estimated Lithium Exposure From Drinking Groundwater

eTable 7. Incidence Rate Ratios and 95% CIs From Poisson Regression for Cancer Risk According to Estimated Lithium Exposure From Drinking Groundwater in the US Study Population, Stratified by Geographic Regions

eTable 8. Hazard Ratios and 95% CIs for Cancer Risk in the US Study Population According to Estimated Lithium Exposure From Drinking Groundwater, Based on 2009-2018 Lithium Measure Data

eTable 9. Hazard Ratios and 95% CIs for Cancer Risk in the US Study Population According to Estimated Lithium Exposure From Drinking Groundwater, Based on 2009-2018 Lithium Measure Data and Stratified by Geographic Regions

eTable 10. Odds Ratios and 95% CIs for Cancer Record at Any Time in the US Study Population From Both Electronic Health Records and Questionnaires, According to Estimated Lithium Exposure From Drinking Groundwater

eTable 11. Odds Ratios and 95% CIs for Cancer Record at Any Time in the US Study Population From Both Electronic Health Records and Questionnaires, According to Estimated Lithium Exposure From Drinking Groundwater Stratified by Western and Eastern States

eTable 12. Hazard Ratios and 95% CIs for Cancer Risk in the US Study Population According to Estimated Lithium Exposure in Areas Where More Than 30% of the Population Uses Drinking Water Supplied by Groundwater

eTable 13. Hazard Ratios and 95% CIs for Cancer Risk in the US Study Population According to Estimated Lithium Exposure From Drinking Groundwater in Areas With Low Arsenic Concentration

This supplementary material has been provided by the authors to give readers additional information about their work.

© 2025 Luo J et al. *JAMA Network Open*.

eMethods 1. Covariates adjusted in this study

We retrieved basic demographic and lifestyle information for each participant from the survey data, including sex at birth (female, male, other), race/ethnicity (non-Hispanic White, non-Hispanic Black, Hispanic, other), age at enrollment, education (less than high school, high school or equivalent, some college, college, graduate), household income (<$35K, $35-50K, $50-75K, $75-150K, >$150K), smoking status (yes, no), and alcohol drinking status (yes, no). Body mass index (BMI; underweight, normal, overweight, obese) was measured at enrollment. We also obtained data on the deprivation index of participants' residential address from All of Us (All of Us Research Program Investigators. The "All of Us" research program. *New England Journal of Medicine*. 2019;381(7):668-676) . The deprivation index is a composite score based on different socioeconomic variables to reflect the overall deprivation level of a community. All these variables were considered as potential confounders in this study.

© 2025 Luo J et al. *JAMA Network Open*.

eMethods 2. Details about kriging for lithium exposure assessment in the contiguous United States

In the study, kriging techniques were employed to estimate the spatial continuous concentrations for lithium. This method computes a weighted average of lithium observations from neighboring well sample sites for each unmonitored grid cell on a map. The weight assigned to each observation is inversely proportional to its distance from the grid point s_0, where the interpolation is required. Generally, the inverse distance interpolator is given as shown in Equation (1):

$$\hat{y}(s_0) = \frac{\sum_{i=1}^{n} w(s_i)\, y(s_i)}{\sum_{i=1}^{n} w(s_i)} \tag{1}$$

where $\hat{y}(s_0)$ is the interpolated value at location s_0, and $y(s_i)$ is the observed value at the ith well sample site for *her* = 1, …, n, with n being the total number of sites available during the exposure time window in the study domain. The weights $w(s_i)$ assigned to unmonitored locations are computed as in Equation (2):

$$w(s_i) = \left|\left|s_i - s_0\right|\right|^{-p} \tag{2}$$

Where, $\left|\left|s_i - s_0\right|\right|$ is the Euclidian distance between location s_i and s_0, p is an inverse-distance weighting power that controls the rate at which weight decreases as zero as the distance increases.

Grid cells of 1 × 1 km and a power of p = 2.2 were applied. The concentration results of lithium were subsequently averaged for each 3-digit Zip code boundaries for various time windows.

© 2025 Luo J et al. *JAMA Network Open*.

eMethods 3. USGS model for groundwater lithium concentration groups

This model was trained using lithium measurements from over 13,500 wells and predictor variables related to its natural occurrence in groundwater. Based on the extreme gradient boosting algorithm, the model predicted the probabilities of lithium concentration in each 1km×1km grid for being one of the following groups: ≤4 μg/L, >4 to ≤10 μg/L, >10 to ≤30 μg/L, and >30 μg/L. The sum of all probabilities across the four groups equals one. These probabilities can be downloaded from the following website: https://www.sciencebase.gov/catalog/item/643a9ad1d34ee8d4ade3b4b3. We averaged the probabilities for the four lithium concentration groups of all 1km×1km grids within each 3-digit zip code area, respectively. The lithium classification for each 3-digit zip code area is based on the lithium concentration group that has the highest probability. The lithium classification was further linked to the lithium concentration group with cancer risks. Results using the USGS lithium classification can be found in the eAppendix.

© 2025 Luo J et al. *JAMA Network Open*.

eMethods 4. Poisson regression and logistic regression models in sensitivity analyses

In the Poisson regression model, we included the population as in the main model. The outcome is also the binary variable for cancer status of each participant at the end of follow-up. Additionally, we added an offset term equal to the natural logarithm of follow-up time of each participant in the regression model. Therefore, the results from the Poisson regression model can be interpreted as the incidence rate ratio (IRR) for cancer risks. The three stratified terms in the main Cox regression model, i.e., sex at birth, race/ethnicity, and age, were treated as conventional covariates in the Poisson regression model, along with other covariates including education, household income, smoking status, alcohol drinking status, and the deprivation index of residential address. To account for residual autocorrelation within the geographic unit, we used the generalized estimating equation (GEE) method to estimate the standard error, with the geographical unit, i.e., 3-digit zip code, as the group factor in GEE.

In the logistic regression model, we included all participants with EHR in *All of Us*. The outcome is binary variable (yes/no) for cancer record in EHR or questionnaire of each participant, no matter before the enrollment or after the enrollment. The model was adjusted for sex at birth, race/ethnicity, age, education, household income, smoking status, alcohol drinking status, and the deprivation index of residential address as conventional covariates. We also used GEE to estimate the standard error to account for residual autocorrelation within the geographic unit.

© 2025 Luo J et al. *JAMA Network Open*.

eMethods 5. Estimate the proportion of population covered by groundwater supply at the 3-digit zip code level

The Drinking Water Mapping Application to Protect Source Waters (DWMAPS) developed by Environmental Protection Agency (EPA) provides the count of drinking water sources from groundwater and surface water and population served by each type of water source at the county level (https://geopub.epa.gov/dwwidgetapp/). We calculated the total number of the population served by each drinking water source, and thus the groundwater percentage. This data was used to estimate the proportion of population covered by groundwater source. In spatial analysis platform, we calculated the overlap percentage between each county and 3-digit zip code areas. The overlap percentage was used as the weight to estimate the weighted proportion of population served by groundwater source at the 3-digit zip code level.

© 2025 Luo J et al. *JAMA Network Open*.

eAppendix. Results using USGS lithium concentration classification based on machine learning model

Exposure level

More than half of the study population living area with lithium concentration classified as ≤4.0 µg/L (eFigure 2). Lithium groups ≤4.0 µg/L and >4 to ≤10 µg/L are more common in the Eastern states, while >10 to ≤30 µg/L and >30 µg/L groups are more common in the Western states.

Full population

In this study, higher lithium exposure was associated with decreased cancer risk, including all cancer types combined, across both males and female, and in both the overall population as well as restricted to long-term residents (eTable 11). In the overall population, compared to the ≤4.0 µg/L group, all other higher exposure groups were associated with a decreased risk for all cancers, with significant association observed for the >30 µg/L group (hazard ratio (HR)=0.10, 95% confidence interval (CI): 0.02-0.45). When restricted to long-term residents, these associations remained almost unchanged; significant association was still observed for the >30 µg/L group (HR=0.13, 95% CI: 0.03-0.54).

The inverse associations with all cancers persisted when the population was stratified into males and females. For the >30 µg/L group, the association was HR=0.11 (95% CI: 0.03-0.44) in females and HR=0.09 (95% CI: 0.02-0.43) in males. When restricted to long-term residents, the associations for the >30 µg/L group were attenuated to HR=0.13 (95% CI: 0.03-0.52) in females and HR=0.12 (95% CI: 0.03-0.57) in males.

© 2025 Luo J et al. *JAMA Network Open*.

The inverse associations were also observed for all individual cancer types (breast, prostate, bladder and urinary, central nervous system (CNS), kidney, colorectal, leukemia, non-Hodgkin's lymphoma (NHL), and thyroid cancer) investigated in this study. Compared to the ≤4.0 µg/L group, all higher groups were associated with decreased risks for all these individual cancer types. The >4 to ≤10 µg/L group exhibited significant associations for breast cancer (HR=0.41, 95% CI: 0.19-0.89), prostate cancer (HR=0.43, 95% CI: 0.21-0.87), bladder cancer (HR=0.34, 95% CI: 0.15-0.77), kidney cancer (HR=0.56, 95% CI: 0.35-0.92), and NHL (HR=0.27, 95% CI: 0.10-0.70). The >30 µg/L group demonstrated stronger associations, with HR ranging from 0.05 to 0.14, significant for all cancer types. Most of the significant associations with >4 to ≤10 µg/L and >30 µg/L groups remained when analysis was restricted to long-term residents, except for kidney cancer and leukemia, which had borderline associations.

© 2025 Luo J et al. *JAMA Network Open*.

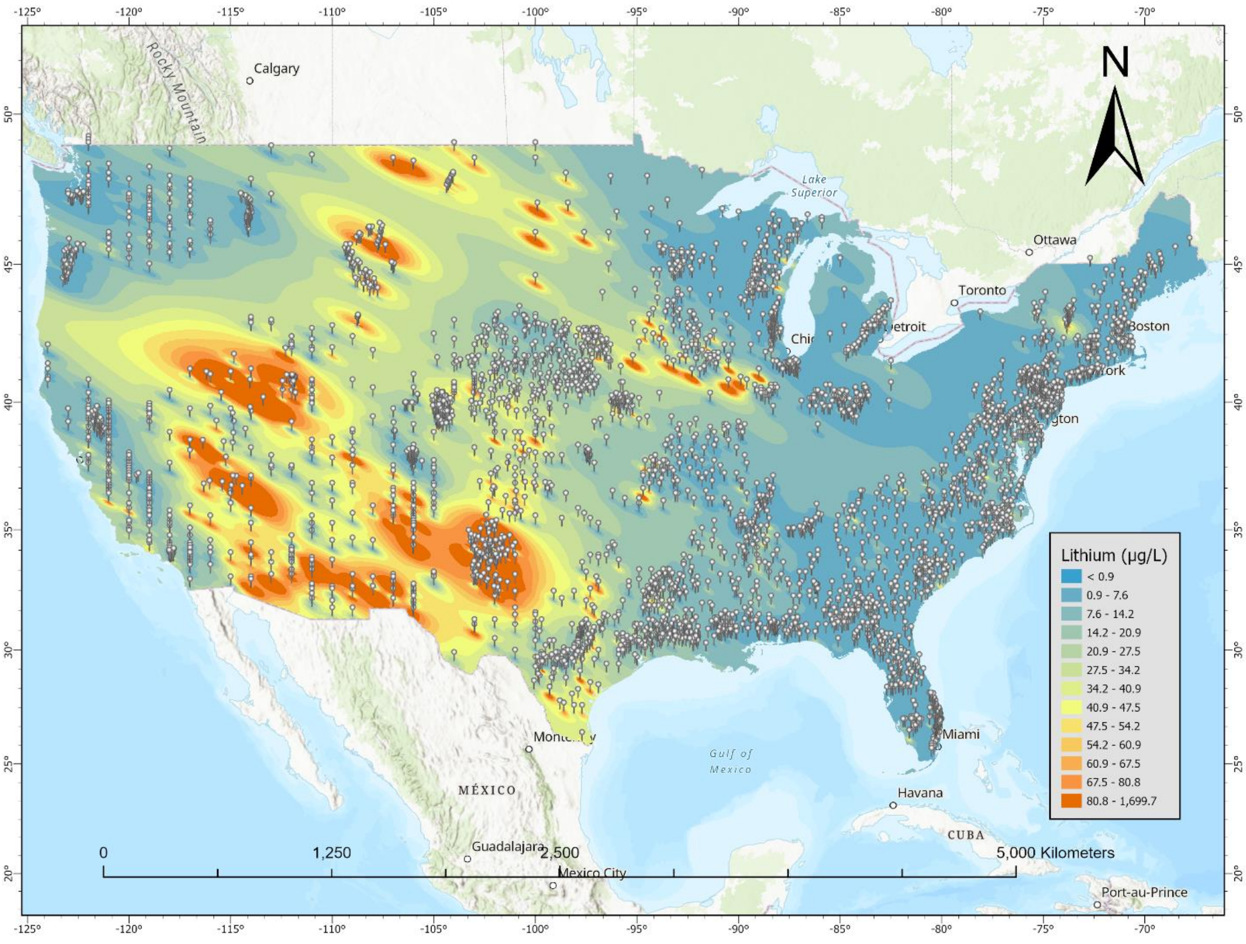

eFigure 1. Spatial distribution of wells for lithium concentration measure and final kriging map of estimated lithium concentration in the contiguous United States.

© 2025 Luo J et al. *JAMA Network Open*.

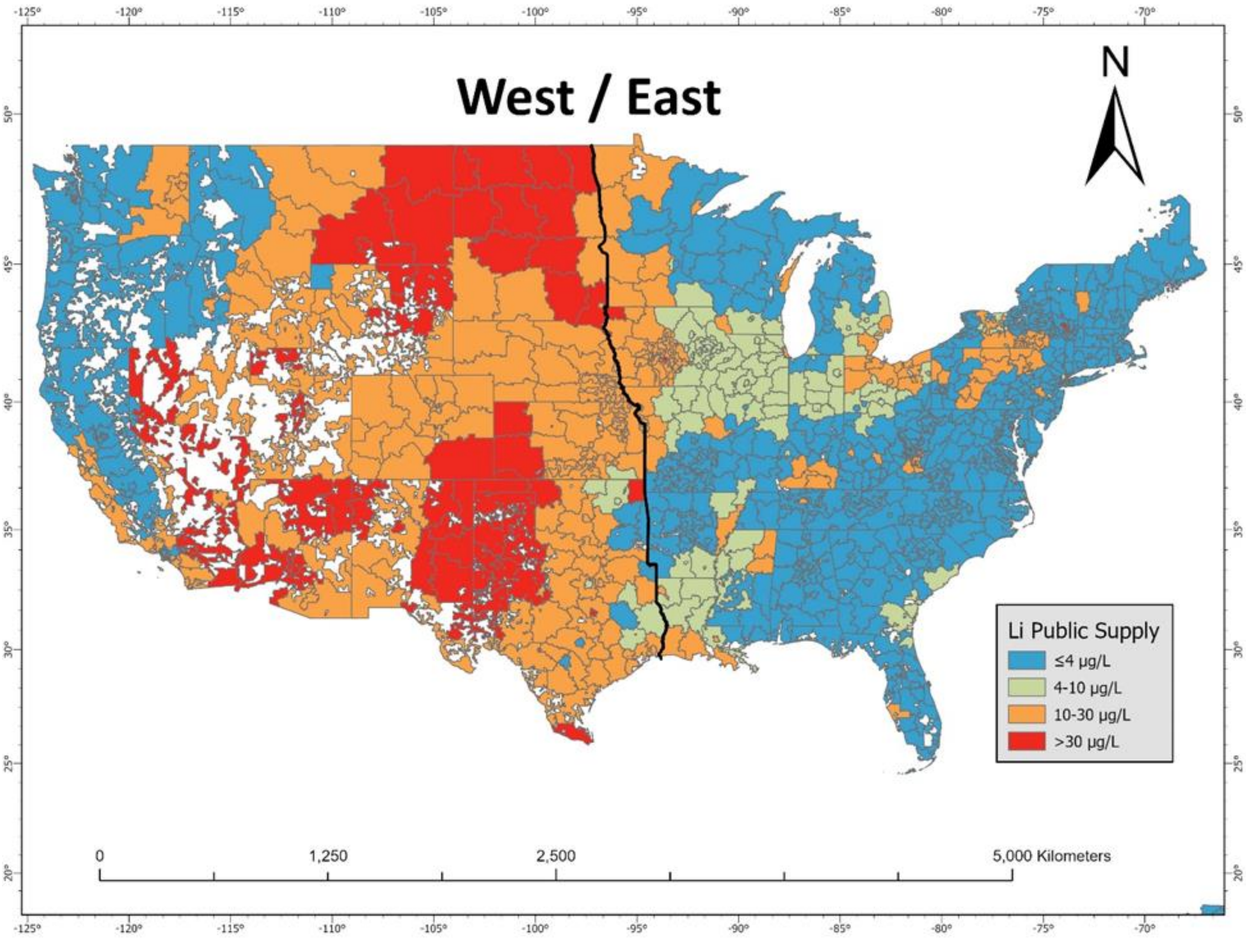

eFigure 2. U.S. Geological Survey **(**USGS) **l**ithium concentration groups for public supply drinking groundwater at the 3-digit zip code level in the contiguous United States.

© 2025 Luo J et al. *JAMA Network Open*.

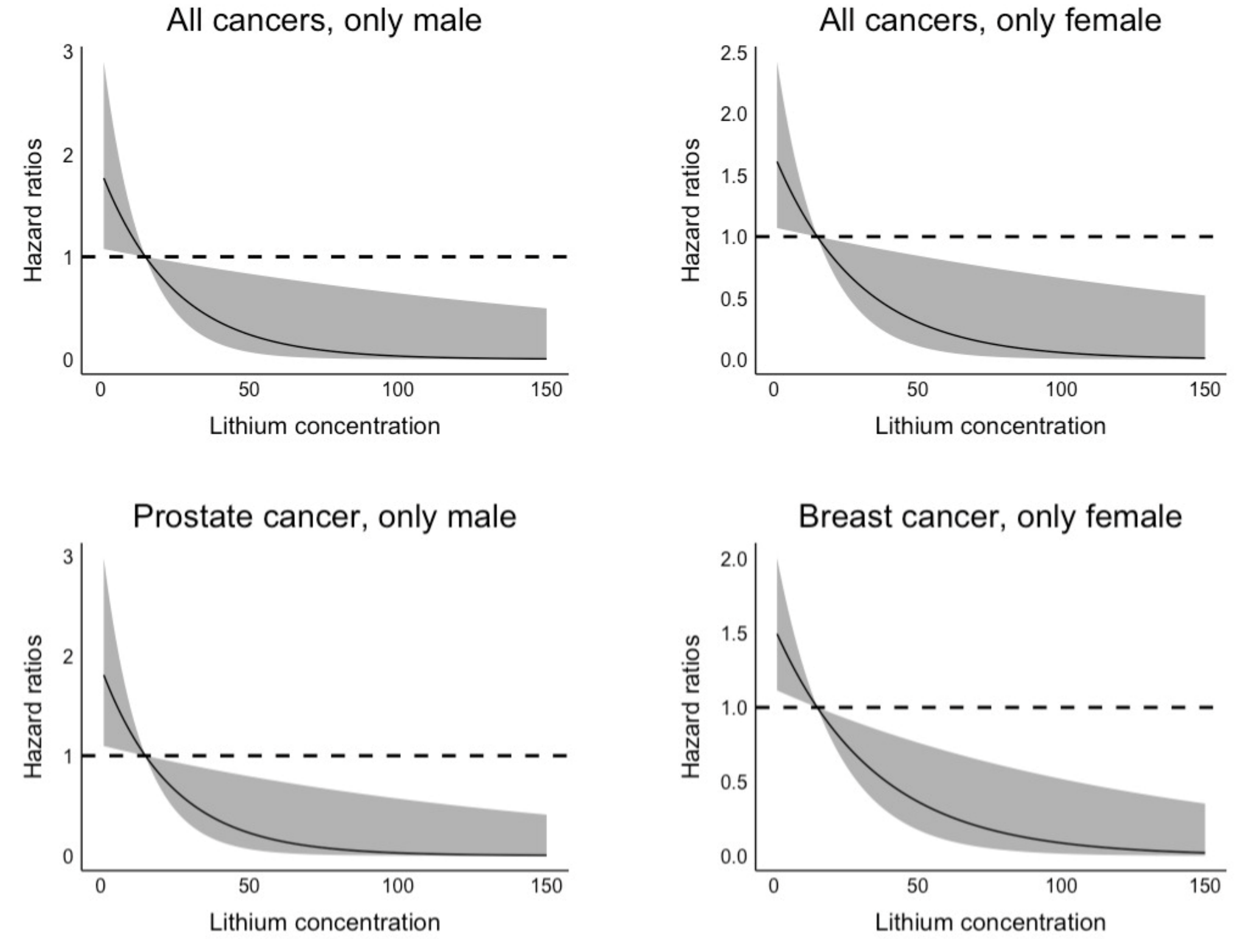

eFigure 3. Non-linear relationship between lithium exposure level in drinking groundwater and cancer outcomes in the United States study population. The shaded area is the 95% confidence interval.

© 2025 Luo J et al. *JAMA Network Open*.

eTable 1. Distribution of estimated lithium exposure from drinking groundwater in the United States study population categorized into 5 groups based on quintile, with the 1st quintile (the lowest) as reference.

Regions	Descriptive statistics (µg/L)						
	Min	Q1	Median	Q3	Max	Mean	SD
Overall	1.3	4.1	7.0	17.8	149.9	16.1	20.0
West	2.4	18.3	43.2	53.6	149.9	39.3	21.6
East	1.3	3.6	5.7	7.1	68.2	5.4	2.7

© 2025 Luo J et al. *JAMA Network Open*.

eTable 2. Hazard ratio and 95% confidence interval for cancer risk according to estimated lithium exposure from drinking groundwater in population stratified by geographical regions among long-term residents. The estimated lithium exposure was categorized into 5 groups based on the quintile, with the 1st quintile (the lowest) as the reference.

	**Long-term residents**							
**Regional-specific quintile**	**Western states**				**Eastern states**			
	Lithium level (µg/L)	Total number	Cancer cases	HR (95% CI)	Lithium level (µg/L)	Total number	Cancer cases	HR (95% CI)
**All cancers**								
1st quintile	2.4-17.8	10539	472	Ref	1.3-3.4	22704	1550	Ref
2nd quintile	17.9-29.6	8291	353	1.26 (0.66-2.40)	3.4-4.4	22029	819	0.50 (0.28-0.89)
3rd quintile	29.7-44.0	10865	225	0.52 (0.22-1.23)	4.5-6.6	22411	710	0.48 (0.27-0.87)
4th quintile	44.1-65.8	11384	69	0.13 (0.03-0.57)	6.7-7.2	25913	1258	0.68 (0.47-0.97)
5th quintile	65.9-149.9	5269	<20	0.01 (0.00-0.08)	7.3-68.2	18484	508	0.32 (0.20-0.52)
**All cancers, only female**								
1st quintile	2.4-17.8	6213	286	Ref	1.3-3.4	14212	835	Ref
2nd quintile	17.9-29.6	5038	212	1.44 (0.78-2.64)	3.4-4.4	14131	471	0.50 (0.27-0.94)
3rd quintile	29.7-44.0	6579	115	0.43 (0.18-1.02)	4.5-6.6	13896	422	0.54 (0.30-0.99)
4th quintile	44.1-65.8	6946	33	0.10 (0.02-0.50)	6.7-7.2	16679	723	0.65 (0.43-0.98)
5th quintile	65.9-149.9	3070	<20	0.01 (0.00-0.12)	7.3-68.2	11477	298	0.35 (0.21-0.57)
**All cancers, only male**								
1st quintile	2.4-17.8	3640	166	Ref	1.3-3.4	8012	698	Ref
2nd quintile	17.9-29.6	3771	155	0.93 (0.45-1.92)	3.4-4.4	8557	337	0.45 (0.25-0.81)
3rd quintile	29.7-44.0	4180	110	0.67 (0.27-1.66)	4.5-6.6	7378	291	0.49 (0.28-0.85)
4th quintile	44.1-65.8	4337	36	0.15 (0.03-0.72)	6.7-7.2	9097	508	0.69 (0.50-0.95)
5th quintile	65.9-149.9	2154	0	0.00 (0.00-0.00)	7.3-68.2	6815	203	0.29 (0.17-0.50)
**Breast cancer, only female**								
1st quintile	2.4-17.8	6043	122	Ref	1.3-3.4	14024	275	Ref
2nd quintile	17.9-29.6	4940	101	1.84 (0.99-3.42)	3.4-4.4	13355	154	0.52 (0.29-0.95)
3rd quintile	29.7-44.0	6476	37	0.40 (0.17-0.94)	4.5-6.6	13854	140	0.53 (0.27-1.02)
4th quintile	44.1-65.8	6925	<20	0.10 (0.02-0.42)	6.7-7.2	15877	244	0.70 (0.47-1.05)
5th quintile	65.9-149.9	3070	<20	0.03 (0.00-0.36)	7.3-68.2	11259	94	0.33 (0.19-0.59)
**Prostate cancer, only male**								
1st quintile	2.4-17.8	3556	44	Ref	1.3-3.4	7766	220	Ref
2nd quintile	17.9-29.6	3603	33	0.78 (0.29-2.09)	3.4-4.4	8060	101	0.43 (0.24-0.77)
3rd quintile	29.7-44.0	4104	37	0.99 (0.40-2.47)	4.5-6.6	7253	85	0.43 (0.23-0.81)
4th quintile	44.1-65.8	4308	<20	0.12 (0.02-0.75)	6.7-7.2	8642	167	0.73 (0.50-1.07)
5th quintile	65.9-149.9	2154	0	0.00 (0.00-0.00)	7.3-68.2	6674	67	0.29 (0.16-0.53)

© 2025 Luo J et al. *JAMA Network Open*.

eTable 3. Hazard ratio and 95% confidence interval for cancer risk in the United States study population stratified by population density

Group-specific tertile	Population density groups											
	Low (1st tertile)				Medium (2nd tertile)				High (3rd tertile)			
	Li level (µg/L)	Total #	Cancer cases	HR (95% CI)	Li level (µg/L)	Total #	Cancer cases	HR (95% CI)	Li level (µg/L)	Total #	Cancer cases	HR (95% CI)
**All cancers**												
1st tertile	1.4-8.6	30233	1073	Ref	1.3-4.4	30088	1159	Ref	1.4-4.1	28370	1597	Ref
2nd tertile	87-45.9	38396	710	0.60 (0.28-1.32)	4.5-7.2	24189	697	0.59 (0.38-0.93)	4.2-7.0	37617	1150	0.58 (0.30-1.13)
3rd tertile	46.0-109.2	21973	61	0.08 (0.01-0.67)	7.3-149.9	26124	862	0.80 (0.51-1.25)	7.1-70.8	15180	260	0.31 (0.14-0.67)
**All cancers, only female**												
1st tertile	1.4-8.6	17509	635	Ref	1.3-4.4	19853	694	Ref	1.4-4.1	16721	857	Ref
2nd tertile	87-45.9	22450	351	0.51 (0.23-1.10)	4.5-7.2	15739	386	0.57 (0.36-0.90)	4.2-7.0	21330	618	0.61 (0.30-1.25)
3rd tertile	46.0-109.2	13222	34	0.08 (0.01-0.57)	7.3-149.9	15344	565	0.55 (0.34-0.90)	7.1-70.8	9410	156	0.35 (0.16-0.76)
**All cancers, only male**												
1st tertile	1.4-8.6	12074	420	Ref	1.3-4.4	9760	442	Ref	1.4-4.1	11070	716	Ref
2nd tertile	87-45.9	15148	353	0.77 (0.34-1.71)	4.5-7.2	7907	297	0.76 (0.47-1.21)	4.2-7.0	15316	497	0.54 (0.29-1.02)
3rd tertile	46.0-109.2	8421	27	0.09 (0.01-0.82)	7.3-149.9	10344	284	0.66 (0.42-1.03)	7.1-70.8	5456	98	0.26 (0.12-0.57)
**Breast cancer, only female**												
1st tertile	1.4-8.6	17056	230	Ref	1.3-4.4	19581	233	Ref	1.4-4.1	16061	268	Ref
2nd tertile	87-45.9	22222	141	0.58 (0.27-1.26)	4.5-7.2	15210	220	1.03 (0.63-1.68)	4.2-7.0	20871	203	0.61 (0.30-1.24)
3rd tertile	46.0-109.2	13197	<20	0.07 (0.01-0.48)	7.3-149.9	14998	124	0.49 (0.27-0.88)	7.1-70.8	9283	42	0.29 (0.13-0.65)
**Prostate cancer, only male**												
1st tertile	1.4-8.6	11761	126	Ref	1.3-4.4	9558	98	Ref	1.4-4.1	10526	195	Ref
2nd tertile	87-45.9	14886	97	0.82 (0.34-1.99)	4.5-7.2	7584	140	1.35 (0.78-2.36)	4.2-7.0	14943	140	0.54 (0.28-1.01)
3rd tertile	46.0-109.2	8400	<20	0.08 (0.01-0.79)	7.3-149.9	10111	62	0.48 (0.25-0.95)	7.1-70.8	5386	33	0.33 (0.14-0.79)

© 2025 Luo J et al. *JAMA Network Open*.

eTable 4. Results based on U.S. Geological Survey (USGS) lithium concentration group model for the United States study population.

Lithium concentration group	Full population			Long-term residents[a]		
	Total number	Cancer cases	HR (95% CI)[b]	Total number	Cancer cases	HR (95% CI)[b]
**All cancers**						
≤4.0 µg/L	133281	4959	Ref	87459	3976	Ref
>4 to ≤10 µg/L	23575	491	0.50 (0.25-1.02)	15080	376	0.49 (0.24-1.02)
>10 to ≤30 µg/L	65466	2025	0.79 (0.56-1.12)	40553	1536	0.82 (0.58-1.17)
>30 µg/L	29856	94	0.10 (0.02-0.45)	14797	77	0.13 (0.03-0.54)
**All cancers, only female**						
≤4.0 µg/L	82452	2847	Ref	55976	2285	Ref
>4 to ≤10 µg/L	13631	275	0.51 (0.25-1.06)	8959	211	0.51 (0.25-1.06)
>10 to ≤30 µg/L	38003	1118	0.80 (0.55-1.15)	24413	856	0.84 (0.58-1.21)
>30 µg/L	17498	56	0.11 (0.03-0.44)	8893	44	0.13 (0.03-0.52)
**All cancers, only male**						
≤4.0 µg/L	48192	2030	Ref	30580	1651	Ref
>4 to ≤10 µg/L	9397	205	0.52 (0.27-1.01)	5961	161	0.48 (0.23-0.99)
>10 to ≤30 µg/L	26015	861	0.91 (0.60-1.39)	15625	659	0.80 (0.56-1.15)
>30 µg/L	11894	38	0.09 (0.02-0.43)	5775	33	0.12 (0.03-0.57)
**Breast cancer, only female**						
≤4.0 µg/L	80398	969	Ref	54353	797	Ref
>4 to ≤10 µg/L	13420	79	0.41 (0.19-0.89)	8797	61	0.40 (0.18-0.91)
>10 to ≤30 µg/L	37208	404	0.81 (0.54-1.21)	23809	306	0.83 (0.56-1.22)
>30 µg/L	17459	20	0.11 (0.03-0.40)	8864	<20	0.13 (0.03-0.52)
**Prostate cancer, only male**						
≤4.0 µg/L	46696	602	Ref	29397	519	Ref
>4 to ≤10 µg/L	9241	54	0.43 (0.21-0.87)	5843	46	0.42 (0.19-0.93)
>10 to ≤30 µg/L	25357	234	0.83 (0.52-1.32)	15132	190	0.73 (0.48-1.12)
>30 µg/L	11863	<20	0.07 (0.01-0.41)	5748	<20	0.08 (0.01-0.49)
**Bladder and urinary cancer**						
≤4.0 µg/L	128215	145	Ref	83414	119	Ref
>4 to ≤10 µg/L	23073	<20	0.34 (0.15-0.77)	14698	<20	0.36 (0.15-0.86)
>10 to ≤30 µg/L	63371	44	0.60 (0.36-1.01)	38977	39	0.72 (0.43-1.20)
>30 µg/L	29761	<20	0.07 (0.01-0.63)	14721	<20	0.10 (0.01-0.89)
**CNS cancer**						
≤4.0 µg/L	128153	83	Ref	83355	60	Ref
>4 to ≤10 µg/L	23070	<20	0.40 (0.15-1.10)	14692	<20	0.25 (0.08-0.79)
>10 to ≤30 µg/L	63363	36	0.91 (0.54-1.52)	38959	21	0.77 (0.43-1.36)
>30 µg/L	29760	<20	0.05 (0.01-0.46)	14720	<20	0.08 (0.01-0.78)
**Colorectal cancer**						
≤4.0 µg/L	128256	186	Ref	83439	144	Ref
>4 to ≤10 µg/L	23081	<20	0.52 (0.24-1.12)	14702	<20	0.45 (0.21-0.99)
>10 to ≤30 µg/L	63415	88	0.88 (0.56-1.37)	39004	66	0.88 (0.55-1.41)
>30 µg/L	29764	<20	0.14 (0.02-0.78)	14722	<20	0.13 (0.03-0.59)
**Kidney cancer**						
≤4.0 µg/L	89949	117	Ref	82765	82	Ref
>4 to ≤10 µg/L	48416	<20	0.56 (0.35-0.92)	18416	<20	0.64 (0.40-1.03)
>10 to ≤30 µg/L	57918	57	0.75 (0.46-1.22)	37218	36	0.74 (0.47-1.17)
>30 µg/L	48137	<20	0.11 (0.03-0.40)	13137	<20	0.20 (0.04-1.03)
**Leukemia**						

© 2025 Luo J et al. *JAMA Network Open*.

≤4.0 μg/L	128211	141	Ref	83410	115	Ref
>4 to ≤10 μg/L	23078	<20	0.57 (0.27-1.22)	14700	<20	0.49 (0.21-1.17)
>10 to ≤30 μg/L	63386	59	0.76 (0.46-1.25)	38983	45	0.76 (0.47-1.22)
>30 μg/L	29764	<20	0.14 (0.02-0.84)	14724	<20	0.20 (0.04-1.15)
**NHL**						
≤4.0 μg/L	128292	222	Ref	83469	174	Ref
>4 to ≤10 μg/L	23074	<20	0.27 (0.10-0.70)	14700	<20	0.34 (0.13-0.89)
>10 to ≤30 μg/L	63409	82	0.71 (0.43-1.18)	39005	67	0.79 (0.49-1.26)
>30 μg/L	29764	<20	0.11 (0.03-0.48)	14722	<20	0.10 (0.02-0.46)
**Thyroid cancer**						
≤4.0 μg/L	128336	266	Ref	83505	210	Ref
>4 to ≤10 μg/L	23082	<20	0.44 (0.18-1.07)	14706	<20	0.50 (0.20-1.29)
>10 to ≤30 μg/L	63396	69	0.55 (0.33-0.91)	38985	47	0.51 (0.28-0.92)
>30 μg/L	29761	<20	0.04 (0.00-0.37)	14721	<20	0.06 (0.01-0.55)

CNS, central nervous system; HR, hazard ratio; NHL, non-Hodgkin's lymphoma; CI, confidence interval

Per *All of Us* Research Program policy, cells with frequency <20 will not be presented.

[a] Long-term residents referred to participants who reported they have lived in the current address for at least 3 years.

[b] Three stratified terms were included: sex at birth, race/ethnicity, and age. Adjusted for education, household income, smoking status, alcohol drinking status, and the deprivation index of residential address.

© 2025 Luo J et al. *JAMA Network Open*.

eTable 5. Hazard ratio and 95% confidence interval for cancer risk in the United States study population without lithium medication history. The estimated lithium exposure was categorized into 5 groups based on the quintile, with the 1st quintile (the lowest) as the reference.

Lithium concentration group	Population without lithium medication history		
	Total number	Cancer cases	HR (95% CI)[b]
**All cancers**			
1st quintile (1.3-3.6 ug/L)	49392	2239	Ref
2nd quintile (3.7-6.1 ug/L)	45757	1405	0.67 (0.39-1.16)
3rd quintile (6.2-7.2 ug/L)	47620	1782	0.76 (0.52-1.10)
4th quintile (7.3-21.3 ug/L)	43594	1237	0.55 (0.36-0.86)
5th quintile (21.4-149.9 ug/L)	46547	462	0.24 (0.11-0.53)
**All cancers, only female**			
1st quintile (1.3-3.6 ug/L)	30360	1209	Ref
2nd quintile (3.7-6.1 ug/L)	27944	805	0.70 (0.39-1.25)
3rd quintile (6.2-7.2 ug/L)	26149	905	0.77 (0.49-1.21)
4th quintile (7.3-21.3 ug/L)	27617	820	0.64 (0.41-1.03)
5th quintile (21.4-149.9 ug/L)	27945	283	0.26 (0.12-0.57)
**All cancers, only male**			
1st quintile (1.3-3.6 ug/L)	18518	995	Ref
2nd quintile (3.7-6.1 ug/L)	17703	586	0.62 (0.37-1.02)
3rd quintile (6.2-7.2 ug/L)	17266	716	0.95 (0.66-1.35)
4th quintile (7.3-21.3 ug/L)	18187	560	0.49 (0.32-0.74)
5th quintile (21.4-149.9 ug/L)	17113	128	0.15 (0.06-0.40)
**Breast cancer, only female**			
1st quintile (1.3-3.6 ug/L)	29462	392	Ref
2nd quintile (3.7-6.1 ug/L)	27340	262	0.68 (0.38-1.23)
3rd quintile (6.2-7.2 ug/L)	25468	296	0.77 (0.48-1.23)
4th quintile (7.3-21.3 ug/L)	27839	314	0.75 (0.44-1.25)
5th quintile (21.4-149.9 ug/L)	26995	103	0.29 (0.13-0.66)
**Prostate cancer, only male**			
1st quintile (1.3-3.6 ug/L)	17489	294	Ref
2nd quintile (3.7-6.1 ug/L)	17243	152	0.52 (0.30-0.89)
3rd quintile (6.2-7.2 ug/L)	17640	253	1.01 (0.73-1.40)
4th quintile (7.3-21.3 ug/L)	16870	125	0.36 (0.22-0.61)
5th quintile (21.4-149.9 ug/L)	17023	38	0.17 (0.06-0.49)

© 2025 Luo J et al. *JAMA Network Open*.

eTable 6. Incidence rate ratio and 95% confidence interval for cancer risk in the United States study population according to estimated lithium exposure from drinking groundwater from Poisson regression. The estimated lithium exposure was categorized into 5 groups based on the quintile, with the 1st quintile (the lowest) as the reference.

	**Full population**		
	Total number	Cancer cases	IRR (95% CI)
**All cancers**			
1st quintile (1.3-3.6 µg/L)	52223	2390	Ref
2nd quintile (3.7-6.1 µg/L)	49235	1497	0.67 (0.62-0.72)
3rd quintile (6.2-7.2 µg/L)	50703	1871	0.72 (0.67-0.77)
4th quintile (7.3-25.5 µg/L)	50955	1510	0.58 (0.54-0.62)
5th quintile (25.6-149.9 µg/L)	49062	301	0.14 (0.13-0.16)

© 2025 Luo J et al. *JAMA Network Open*.

eTable 7. Incidence rate ratio and 95% confidence interval for cancer risk according to estimated lithium exposure from drinking groundwater in the United States study population stratified by geographical regions from Poisson regression. The estimated lithium exposure was categorized into 5 groups based on the quintile, with the 1st quintile (the lowest) as the reference.

Regional-specific quintile	Full population							
	West				East			
	Lithium level (µg/L)	Total number	Cancer cases	IRR (95% CI)	Lithium level (µg/L)	Total number	Cancer cases	IRR (95% CI)
**All cancers**								
1st quintile	2.4-17.8	19214	724	Ref	1.3-3.4	34843	1948	Ref
2nd quintile	17.9-29.6	12535	452	1.02 (0.88-1.19)	3.5-4.4	37202	1011	0.43 (0.40-0.47)
3rd quintile	29.7-44.0	15966	204	0.29 (0.24-0.35)	4.5-6.6	31875	912	0.63 (0.58-0.69)
4th quintile	44.1-65.8	21117	63	0.08 (0.06-0.10)	6.7-7.2	42021	1623	0.62 (0.57-0.67)
5th quintile	65.9-149.9	10343	<20	0.01 (0.00-0.03)	7.3-68.2	27062	629	0.34 (0.31-0.38)

© 2025 Luo J et al. *JAMA Network Open*.

eTable 8. Hazard ratio and 95% confidence interval for cancer risk in the United States study population according to estimated lithium exposure from drinking groundwater based on lithium measure data between 2009-2018. The estimated lithium exposure was categorized into 5 groups based on the quintile, with the 1st quintile (the lowest) as the reference.

	Full population			Long-term residents		
	Total number	Cancer cases	HR (95% CI)	Total number	Cancer cases	HR (95% CI)
**All cancers**						
1st quintile (1.2-4.2 µg/L)	50454	2558	Ref	32644	2028	Ref
2nd quintile (4.3-6.4 µg/L)	50755	1284	0.58 (0.29-1.19)	30593	1017	0.60 (0.36-1.01)
3rd quintile (6.5-8.4 µg/L)	60608	2011	0.51 (0.26-0.98)	41015	1592	0.63 (0.39-0.99)
4th quintile (8.5-28.9 µg/L)	41905	1401	0.57 (0.33-0.97)	22122	709	0.52 (0.33-0.81)
5th quintile (29.0-110.7 µg/L)	48456	315	0.46 (0.22-0.97)	31515	619	0.36 (0.17-0.77)
**All cancers, only female**						
1st quintile (1.2-4.2 µg/L)	30440	1447	Ref	20161	1112	Ref
2nd quintile (4.3-6.4 µg/L)	30350	685	0.55 (0.33-0.92)	19226	584	0.64 (0.37-1.09)
3rd quintile (6.5-8.4 µg/L)	37953	1182	0.63 (0.41-0.99)	19887	647	0.58 (0.32-1.04)
4th quintile (8.5-28.9 µg/L)	22604	736	0.68 (0.44-1.07)	19522	705	0.66 (0.43-1.02)
5th quintile (29.0-110.7 µg/L)	30237	246	0.21 (0.08-0.54)	19445	348	0.37 (0.17-0.77)
**All cancers, only male**						
1st quintile (1.2-4.2 µg/L)	19396	1125	Ref	11607	898	Ref
2nd quintile (4.3-6.4 µg/L)	18804	533	0.53 (0.33-0.84)	11624	414	0.48 (0.29-0.79)
3rd quintile (6.5-8.4 µg/L)	21423	765	0.71 (0.44-1.13)	13596	632	0.62 (0.40-0.95)
4th quintile (8.5-28.9 µg/L)	16816	572	0.53 (0.35-0.80)	9669	292	0.39 (0.26-0.57)
5th quintile (29.0-110.7 µg/L)	19059	139	0.15 (0.05-0.41)	11445	268	0.35 (0.16-0.78)
**Breast cancer, only female**						
1st quintile (1.2-4.2 µg/L)	29880	485	Ref	30875	370	Ref
2nd quintile (4.3-6.4 µg/L)	29648	214	0.80 (0.69-0.92)	30397	210	0.65 (0.37-1.15)
3rd quintile (6.5-8.4 µg/L)	36746	388	0.53 (0.30-0.93)	38989	317	0.66 (0.41-1.07)
4th quintile (8.5-28.9 µg/L)	23786	317	0.65 (0.42-1.01)	22053	158	0.63 (0.32-1.24)
5th quintile (29.0-110.7 µg/L)	28425	68	0.82 (0.49-1.36)	30531	149	0.50 (0.24-1.04)
**Prostate cancer, only male**						
1st quintile (1.2-4.2 µg/L)	18867	323	Ref	30785	280	Ref
2nd quintile (4.3-6.4 µg/L)	18790	156	0.51 (0.31-0.85)	30321	134	0.56 (0.33-0.93)
3rd quintile (6.5-8.4 µg/L)	20177	242	0.78 (0.49-1.24)	38879	207	0.62 (0.39-0.99)
4th quintile (8.5-28.9 µg/L)	16721	142	0.44 (0.27-0.70)	21965	70	0.32 (0.20-0.52)
5th quintile (29.0-110.7 µg/L)	18602	34	0.16 (0.05-0.53)	30464	82	0.36 (0.15-0.84)
**Bladder and urinary cancer**						
1st quintile (1.2-4.2 µg/L)	49448	81	Ref	30570	65	Ref
2nd quintile (4.3-6.4 µg/L)	48426	29	0.43 (0.22-0.86)	30217	30	0.54 (0.27-1.08)
3rd quintile (6.5-8.4 µg/L)	57921	60	0.66 (0.34-1.26)	38722	50	0.63 (0.31-1.27)
4th quintile (8.5-28.9 µg/L)	40488	23	0.32 (0.17-0.59)	22011	<20	0.27 (0.12-0.62)
5th quintile (29.0-110.7 µg/L)	48137	<20	0.14 (0.04-0.44)	30290	<20	0.19 (0.07-0.50)
**CNS cancer**						
1st quintile (1.2-4.2 µg/L)	49410	43	Ref	30525	20	Ref
2nd quintile (4.3-6.4 µg/L)	48414	<20	0.69 (0.39-1.23)	30203	<20	0.89 (0.37-2.13)
3rd quintile (6.5-8.4 µg/L)	57891	30	0.77 (0.46-1.28)	38696	24	0.99 (0.52-1.91)
4th quintile (8.5-28.9 µg/L)	40498	33	0.82 (0.51-1.33)	22015	<20	1.11 (0.47-2.57)
5th quintile (29.0-110.7 µg/L)	48133	<20	0.12 (0.03-0.41)	30287	<20	0.40 (0.12-1.28)
**Colorectal cancer**						

© 2025 Luo J et al. *JAMA Network Open*.

1st quintile (1.2-4.2 μg/L)	49461	94	Ref	30571	66	Ref
2nd quintile (4.3-6.4 μg/L)	48451	54	0.69 (0.39-1.23)	30234	47	0.85 (0.45-1.60)
3rd quintile (6.5-8.4 μg/L)	57940	79	0.77 (0.46-1.28)	38738	66	0.83 (0.48-1.45)
4th quintile (8.5-28.9 μg/L)	40526	61	0.82 (0.51-1.33)	22028	30	0.63 (0.35-1.14)
5th quintile (29.0-110.7 μg/L)	48138	<20	0.12 (0.03-0.41)	30296	<20	0.30 (0.12-0.72)
**Kidney cancer**						
1st quintile (1.2-4.2 μg/L)	49449	82	Ref	30558	53	Ref
2nd quintile (4.3-6.4 μg/L)	48416	<20	0.27 (0.14-0.52)	30207	20	0.45 (0.23-0.87)
3rd quintile (6.5-8.4 μg/L)	57918	57	0.56 (0.35-0.92)	38713	41	0.58 (0.34-0.98)
4th quintile (8.5-28.9 μg/L)	40500	35	0.50 (0.31-0.81)	22019	21	0.52 (0.30-0.91)
5th quintile (29.0-110.7 μg/L)	48137	<20	0.11 (0.03-0.40)	30290	<20	0.22 (0.09-0.56)
**Leukemia**						
1st quintile (1.2-4.2 μg/L)	49428	61	Ref	30552	47	Ref
2nd quintile (4.3-6.4 μg/L)	48426	29	0.55 (0.28-1.08)	30210	23	0.56 (0.27-1.14)
3rd quintile (6.5-8.4 μg/L)	57912	51	0.78 (0.46-1.33)	38716	44	0.83 (0.45-1.51)
4th quintile (8.5-28.9 μg/L)	40525	60	1.19 (0.68-2.07)	22037	39	1.17 (0.61-2.25)
5th quintile (29.0-110.7 μg/L)	48148	<20	0.31 (0.10-0.98)	30302	23	0.47 (0.20-1.12)
**NHL**						
1st quintile (1.2-4.2 μg/L)	49481	114	Ref	30584	79	Ref
2nd quintile (4.3-6.4 μg/L)	48447	50	0.51 (0.30-0.88)	30233	46	0.66 (0.37-1.18)
3rd quintile (6.5-8.4 μg/L)	57939	78	0.63 (0.36-1.09)	38736	64	0.69 (0.39-1.20)
4th quintile (8.5-28.9 μg/L)	40525	60	0.66 (0.40-1.10)	22034	36	0.68 (0.39-1.17)
5th quintile (29.0-110.7 μg/L)	48147	<20	0.19 (0.06-0.62)	30309	30	0.42 (0.17-1.04)
**Thyroid cancer**						
1st quintile (1.2-4.2 μg/L)	49523	156	Ref	30626	121	Ref
2nd quintile (4.3-6.4 μg/L)	48448	51	0.36 (0.21-0.62)	30235	48	0.41 (0.22-0.78)
3rd quintile (6.5-8.4 μg/L)	57948	87	0.55 (0.30-1.00)	38730	58	0.43 (0.22-0.83)
4th quintile (8.5-28.9 μg/L)	40516	51	0.42 (0.26-0.69)	22028	30	0.38 (0.19-0.76)
5th quintile (29.0-110.7 μg/L)	48140	<20	0.10 (0.03-0.28)	30298	<20	0.19 (0.08-0.47)

© 2025 Luo J et al. *JAMA Network Open*.

eTable 9. Hazard ratio and 95% confidence interval for cancer risk in the United States study population according to estimated lithium exposure from drinking groundwater based on lithium measure data between 2009 and 2018 in population stratified by geographical regions. The estimated lithium exposure was categorized into 5 groups based on the quintile, with the 1st quintile (the lowest) as the reference.

Regional-specific quintile	Full population							
	West				East			
	Lithium level (µg/L)	Total number	Cancer cases	HR (95% CI)	Lithium level (µg/L)	Total number	Cancer cases	HR (95% CI)
**All cancers**								
1st quintile	3.7-16.0	17676	474	Ref	1.2-3.6	35066	2122	Ref
2nd quintile	16.1-29.3	14474	700	1.67 (1.15-2.43)	3.7-4.6	34187	1018	0.45 (0.28-0.73)
3rd quintile	29.4-45.3	15888	208	0.44 (0.14-1.39)	4.7-6.9	35580	755	0.42 (0.23-0.75)
4th quintile	45.4-69.7	20972	63	0.11 (0.02-0.77)	7.0-7.5	33754	1216	0.54 (0.35-0.85)
5th quintile	69.8-110.7	10165	<20	0.01 (0.00-0.06)	7.6-70.6	34416	1012	0.41 (0.30-0.56)
**All cancers, only female**								
1st quintile	3.7-16.0	15808	997	Ref	1.2-3.6	21967	1254	Ref
2nd quintile	16.1-29.3	17513	685	0.64 (0.41-1.01)	3.7-4.6	19970	476	0.38 (0.22-0.65)
3rd quintile	29.4-45.3	15068	425	0.52 (0.30-0.88)	4.7-6.9	22194	440	0.42 (0.23-0.77)
4th quintile	45.4-69.7	20308	585	0.36 (0.21-0.63)	7.0-7.5	20232	656	0.51 (0.33-0.81)
5th quintile	69.8-110.7	9849	250	0.31 (0.19-0.53)	7.6-70.6	20456	634	0.49 (0.36-0.65)
**All cancers, only male**								
1st quintile	3.7-16.0	6680	204	Ref	1.2-3.6	12901	882	Ref
2nd quintile	16.1-29.3	5760	271	1.26 (0.81-1.97)	3.7-4.6	13026	488	0.49 (0.29-0.81)
3rd quintile	29.4-45.3	6198	91	0.42 (0.13-1.32)	4.7-6.9	12757	286	0.35 (0.20-0.61)
4th quintile	45.4-69.7	8193	28	0.09 (0.01-0.72)	7.0-7.5	20580	764	0.59 (0.40-0.87)
5th quintile	69.8-110.7	4196	0	0.00 (0.00-0.00)	7.6-70.6	5207	120	0.31 (0.17-0.57)
**Breast cancer, only female**								
1st quintile	3.7-16.0	9250	73	Ref	1.2-3.6	21024	408	Ref
2nd quintile	16.1-29.3	9478	222	2.85 (1.94-4.18)	3.7-4.6	23515	166	0.35 (0.21-0.61)
3rd quintile	29.4-45.3	9292	44	0.70 (0.25-1.97)	4.7-6.9	17977	133	0.49 (0.24-0.99)
4th quintile	45.4-69.7	12418	11	0.11 (0.02-0.70)	7.0-7.5	19732	214	0.55 (0.34-0.87)
5th quintile	69.8-110.7	5811	0	0.00 (0.00-0.00)	7.6-70.6	19988	201	0.46 (0.33-0.63)
**Prostate cancer, only male**								
1st quintile	3.7-16.0	6508	36	Ref	1.2-3.6	12737	267	Ref
2nd quintile	16.1-29.3	5726	70	1.60 (0.83-3.09)	3.7-4.6	12339	120	0.40 (0.24-0.67)
3rd quintile	29.4-45.3	9658	27	0.69 (0.16-3.07)	4.7-6.9	12708	101	0.39 (0.22-0.70)
4th quintile	45.4-69.7	4471	<20	0.21 (0.02-2.31)	7.0-7.5	19693	232	0.59 (0.39-0.89)
5th quintile	69.8-110.7	4196	0	0.00 (0.00-0.00)	7.6-70.6	5121	38	0.31 (0.17-0.58)

© 2025 Luo J et al. *JAMA Network Open*.

eTable 10. Odds ratio and 95% confidence interval for cancer record in the United States study population from both electronic health record (EHR) and questionnaire at any time according to estimated lithium exposure from drinking groundwater. The estimated lithium exposure was categorized into 5 groups based on the quintile, with the 1st quintile (the lowest) as the reference.

	Population with cancer record			Long-term residents with cancer record		
	Total number	Cancer cases	OR (95% CI)	Total number	Cancer cases	OR (95% CI)
**All cancers**						
1st quintile (1.3-3.6 µg/L)	61149	11316	Ref	41583	9362	Ref
2nd quintile (3.7-6.1 µg/L)	57450	9712	0.84 (0.81-0.87)	34065	7329	0.83 (0.80-0.87)
3rd quintile (6.2-7.2 µg/L)	57859	9027	0.77 (0.74-0.80)	38272	7121	0.79 (0.76-0.83)
4th quintile (7.3-23.1 µg/L)	55620	9847	0.75 (0.72-0.78)	37063	7568	0.73 (0.70-0.76)
5th quintile (23.2- 149.9 µg/L)	61149	5346	0.46 (0.45-0.48)	37715	5394	0.56 (0.54-0.59)
**All cancers, only female**						
1st quintile (1.3-3.6 µg/L)	37310	6549	Ref	26309	5453	Ref
2nd quintile (3.7-6.1 µg/L)	34904	5790	0.85 (0.81-0.89)	20746	4260	0.84 (0.80-0.89)
3rd quintile (6.2-7.2 µg/L)	34954	5595	0.78 (0.74-0.81)	24514	4571	0.81 (0.77-0.86)
4th quintile (7.3-23.1 µg/L)	32201	5468	0.79 (0.75-0.82)	22264	4376	0.76 (0.72-0.80)
5th quintile (23.2- 149.9 µg/L)	34667	3346	0.50 (0.48-0.53)	22799	3127	0.58 (0.54-0.61)
**All cancers, only male**						
1st quintile (1.3-3.6 µg/L)	22714	4540	Ref	14894	3829	Ref
2nd quintile (3.7-6.1 µg/L)	21261	3684	0.81 (0.76-0.85)	13821	3130	0.79 (0.74-0.85)
3rd quintile (6.2-7.2 µg/L)	21954	3305	0.75 (0.71-0.80)	13593	2660	0.72 (0.67-0.77)
4th quintile (7.3-23.1 µg/L)	22928	4386	0.72 (0.68-0.76)	14013	3056	0.66 (0.62-0.70)
5th quintile (23.2- 149.9 µg/L)	21015	1593	0.38 (0.35-0.41)	13766	1975	0.49 (0.46-0.53)

© 2025 Luo J et al. *JAMA Network Open*.

eTable 11. Odds ratio and 95% confidence interval for cancer record in the United States study population stratified by Western and Eastern states from both electronic health record (EHR) and questionnaire at any time according to estimated lithium exposure from drinking groundwater. The estimated lithium exposure was categorized into 5 groups based on the quintile, with the 1st quintile (the lowest) as the reference.

	**Population with history record**							
	**Western states**				**Eastern states**			
**Regional-specific quintile**	Lithium level (µg/L)	Total number	Cancer cases	HR (95% CI)	Lithium level (µg/L)	Total number	Cancer cases	HR (95% CI)
**All cancers**								
1st quintile	2.4-17.5	18275	3674	Ref	1.3-3.0	40257	8702	Ref
2nd quintile	17.6-25.5	18296	3617	1.07 (1.01-1.14)	3.1-4.4	43856	6325	0.54 (0.52-0.56)
3rd quintile	25.6-43.2	17477	1777	0.53 (0.49-0.57)	4.5-6.4	36509	6338	0.69 (0.66-0.73)
4th quintile	43.3-65.8	23829	1420	0.35 (0.33-0.38)	6.5-7.2	48409	7219	0.63 (0.60-0.66)
5th quintile	65.9-149.9	11201	861	0.48 (0.44-0.52)	7.3-68.2	31748	5315	0.49 (0.46-0.51)
**All cancers, only female**								
1st quintile	2.4-17.5	10728	2075	Ref	1.3-3.0	24388	5057	Ref
2nd quintile	17.6-25.5	10922	2030	1.12 (1.03-1.22)	3.1-4.4	24514	3375	0.56 (0.53-0.59)
3rd quintile	25.6-43.2	10223	1068	0.52 (0.48-0.58)	4.5-6.4	24083	4053	0.67 (0.63-0.71)
4th quintile	43.3-65.8	14171	843	0.35 (0.31-0.38)	6.5-7.2	29558	4574	0.63 (0.60-0.67)
5th quintile	65.9-149.9	6434	533	0.49 (0.43-0.55)	7.3-68.2	19015	3140	0.49 (0.46-0.52)
**All cancers, only male**								
1st quintile	2.4-17.5	7204	1517	Ref	1.3-3.0	15081	3498	Ref
2nd quintile	17.6-25.5	7057	1531	1.11 (1.00-1.22)	3.1-4.4	16006	2491	0.55 (0.51-0.59)
3rd quintile	25.6-43.2	6897	678	0.54 (0.48-0.61)	4.5-6.4	13857	2374	0.64 (0.60-0.70)
4th quintile	43.3-65.8	9269	546	0.36 (0.32-0.40)	6.5-7.2	17891	2507	0.64 (0.59-0.69)
5th quintile	65.9-149.9	4591	313	0.48 (0.42-0.55)	7.3-68.2	12019	2053	0.48 (0.44-0.52)

© 2025 Luo J et al. *JAMA Network Open*.

eTable 12. Hazard ratio and 95% confidence interval for cancer risk in the United States study population according to estimated lithium exposure in areas where >30% population use drinking water supplied by groundwater. The estimated lithium exposure was categorized into 5 groups based on the quintile, with the 1st quintile (the lowest) as the reference.

	**Areas where >30% population were covered by ground water**		
	Total number	Cancer cases	HR (95% CI)
**All cancers**			
1st quartile (1.3-4.3 μg/L)	17489	892	Ref
2nd quintile (4.4-6.3 μg/L)	12694	98	0.23 (0.07-0.75)
3rd quintile (6.4-37.1 μg/L)	13529	214	0.44 (0.20-0.94)
4th quintile (37.2-97.6 μg/L)	13951	<20	0.02 (0.00-0.12)
**All cancers, only female**			
1st quartile (1.3-4.3 μg/L)	10210	482	Ref
2nd quintile (4.4-6.3 μg/L)	7807	53	0.24 (0.07-0.83)
3rd quintile (6.4-37.1 μg/L)	7746	95	0.38 (0.18-0.81)
4th quintile (37.2-97.6 μg/L)	8534	<20	0.17 (0.02-1.33)
**All cancers, only male**			
1st quartile (1.3-4.3 μg/L)	5574	396	Ref
2nd quintile (4.4-6.3 μg/L)	5906	44	0.10 (0.03-0.30)
3rd quintile (6.4-37.1 μg/L)	5138	92	0.26 (0.13-0.53)
4th quintile (37.2-97.6 μg/L)	5512	<20	0.00 (0.00-0.04)

© 2025 Luo J et al. *JAMA Network Open*.

eTable 13. Hazard ratio and 95% confidence interval for cancer risk in the United States study population according to estimated lithium exposure from drinking groundwater in areas with low arsenic concentration. The estimated lithium exposure was categorized into 5 groups based on the quintile, with the 1st quintile (the lowest) as the reference.

	**Areas with low arsenic concentration**		
	Total number	Cancer cases	HR (95% CI)
**All cancers**			
1st quintile (1.3-4.3 µg/L)	25817	1092	Ref
2nd quintile (4.4-6.1 µg/L)	26714	514	0.77 (0.32-1.88)
3rd quintile (6.2-7.0 µg/L)	27090	850	1.22 (0.62-2.42)
4th quintile (7.1-73.0 µg/L)	20864	980	1.93 (0.90-4.13)
5th quintile (73.1- 149.9 µg/L)	24908	464	0.52 (0.27-0.98)
**All cancers, only female**			
1st quintile (1.3-4.3 µg/L)	15650	615	Ref
2nd quintile (4.4-6.1 µg/L)	15991	316	0.88 (0.38-2.05)
3rd quintile (6.2-7.0 µg/L)	15217	466	1.30 (0.67-2.54)
4th quintile (7.1-73.0 µg/L)	13848	601	1.94 (0.89-4.22)
5th quintile (73.1- 149.9 µg/L)	14378	257	0.56 (0.32-0.98)
**All cancers, only male**			
1st quintile (1.3-4.3 µg/L)	9611	460	Ref
2nd quintile (4.4-6.1 µg/L)	10092	185	0.60 (0.23-1.58)
3rd quintile (6.2-7.0 µg/L)	11174	357	1.10 (0.53-2.28)
4th quintile (7.1-73.0 µg/L)	7888	371	1.27 (0.55-2.96)
5th quintile (73.1- 149.9 µg/L)	8715	196	0.52 (0.27-0.99)
**Breast cancer, only female**			
1st quintile (1.3-4.3 µg/L)	15201	209	
2nd quintile (4.4-6.1 µg/L)	15756	112	0.90 (0.37-2.20)
3rd quintile (6.2-7.0 µg/L)	14874	163	1.42 (0.72-2.80)
4th quintile (7.1-73.0 µg/L)	13401	190	1.92 (0.85-4.37)
5th quintile (73.1- 149.9 µg/L)	14205	96	0.62 (0.31-1.26)
**Prostate cancer, only male**			
1st quintile (1.3-4.3 µg/L)	9270	135	Ref
2nd quintile (4.4-6.1 µg/L)	9956	60	0.62 (0.22-1.70)
3rd quintile (6.2-7.0 µg/L)	10917	113	1.22 (0.59-2.54)
4th quintile (7.1-73.0 µg/L)	7621	120	1.23 (0.52-2.92)
5th quintile (73.1- 149.9 µg/L)	8569	54	0.46 (0.21-0.99)

© 2025 Luo J et al. *JAMA Network Open*.
