# Supplementary material for: Cancer Risk and Estimated Lithium Exposure in Drinking Groundwater in the US
Source: JAMA Netw Open. 2025 Feb 20;8(2):e2460854. doi: 10.1001/jamanetworkopen.2024.60854 (PMC11843356; doi:10.1001/jamanetworkopen.2024.60854)
Supplement: Supplement 2. — Data Sharing Statement [file jamanetwopen-e2460854-s002.pdf]

Data Sharing Statement

Luo. Cancer Risk and Estimated Lithium Exposure in Drinking Groundwater in the US. *JAMA Netw Open*. Published February 20, 2025. doi:10.1001/jamanetworkopen.2024.60854

Data
Data available: Yes
Data types: Deidentified participant data
How to access data: All data and codes will be provided on the All of Us Researcher Workbench for all registered users.
When available: With publication

Supporting Documents
Document types: Statistical/analytic code
How to access documents: All data and codes will be provided on the All of Us Researcher Workbench for all registered users.
When available: With publication

Additional Information
Who can access the data: Registered users of the All of Us Researcher Workbench
Types of analyses: for any purpose
Mechanisms of data availability: Approval of the All of Us Advisory Committee.
